# Recent progress on magnetic iron oxide nanoparticles: synthesis, surface functional strategies and biomedical applications

**DOI:** 10.1088/1468-6996/16/2/023501

**Published:** 2015-04-28

**Authors:** Wei Wu, Zhaohui Wu, Taekyung Yu, Changzhong Jiang, Woo-Sik Kim

**Affiliations:** 1 Laboratory of Printable Functional Nanomaterials and Printed Electronics, School of Printing and Packaging, Wuhan University, Wuhan 430072, People’s Republic of China; 2 Key Laboratory of Artificial Micro- and Nano-structures of Ministry of Education, School of Physics and Technology, Wuhan University, Wuhan 430072, People’s Republic of China; 3 Department of Chemical Engineering, Kyung Hee University, Korea

**Keywords:** magnetic iron oxide nanoparticles, surface functional strategy, biomedical application

## Abstract

This review focuses on the recent development and various strategies in the preparation, microstructure, and magnetic properties of bare and surface functionalized iron oxide nanoparticles (IONPs); their corresponding biological application was also discussed. In order to implement the practical *in vivo* or *in vitro* applications, the IONPs must have combined properties of high magnetic saturation, stability, biocompatibility, and interactive functions at the surface. Moreover, the surface of IONPs could be modified by organic materials or inorganic materials, such as polymers, biomolecules, silica, metals, etc. The new functionalized strategies, problems and major challenges, along with the current directions for the synthesis, surface functionalization and bioapplication of IONPs, are considered. Finally, some future trends and the prospects in these research areas are also discussed.

## 1. Introduction

Iron oxides are common compounds, which are widespread in nature and can be readily synthesized in the laboratory. Magnetic iron oxides have served humans for centuries, for example, the application of small iron oxide nanoparticles (IONPs) as a contrast agent for *in vitro* diagnostics has been practiced for nearly half a century [1–3]. In the past decade, the synthesis of magnetic IONPs has been intensively developed not only for its fundamental scientific interest but also for its many technological applications, such as targeted drug delivery, magnetic resonance imaging (MRI), magnetic hyperthermia and thermoablation, bioseparation, and biosensing [4–7]. Particularly, bioapplications based on magnetic nanoparticles (NPs) have received considerable attention because NPs offer unique advantages over other materials. For example, magnetic IONPs are inexpensive to produce, physically and chemically stable, biocompatible, and environmentally safe [8].

### 1.1. Iron oxides

Eight iron oxides are known [9], among these iron oxides, hematite (*α*-Fe_2_O_3_), magnetite (Fe_3_O_4_) and maghemite (*γ*-Fe_2_O_3_) are very promising and popular candidates due to their polymorphism involving temperature-induced phase transition. Each of these three iron oxides has unique biochemical, magnetic, catalytic, and other properties which provide suitability for specific technical and biomedical applications.

#### 1.1.1. Hematite (*α*-Fe_2_O_3_)

**Figure 1.**
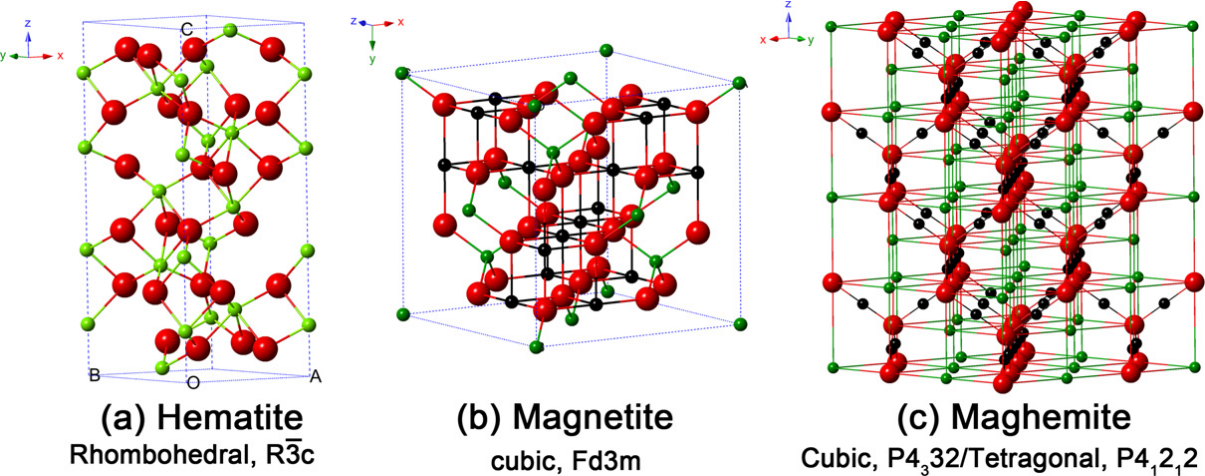
Crystal structure and crystallographic data of the hematite, magnetite and maghemite (the black ball is Fe^2+^, the green ball is Fe^3+^ and the red ball is O^2−^).

As the most stable iron oxide and *n*-type semiconductor under ambient conditions, hematite (*α*-Fe_2_O_3_) is widely used in catalysts, pigments and gas sensors due to its low cost and high resistance to corrosion. It can also be used as a starting material for the synthesis of magnetite (Fe_3_O_4_) and maghemite (*γ*-Fe_2_O_3_), which have been intensively pursued for both fundamental scientific interests and technological applications in the last few decades [10]. Hematite is an *n*-type semiconductor with a band gap of 2.3 eV, where the conduction band (CB) is composed of empty d-orbitals of Fe^3+^ and the valence band (VB) consists of occupied 3d crystal field orbitals of Fe^3+^ with some admixture from the O 2p non-bonding orbitals [11]. As shown in figure 1(a), Fe^3+^ ions occupy two-thirds of the octahedral sites that are confined by the nearly ideal hexagonal close-packed O lattice.

#### 1.1.2. Magnetite (Fe_3_O_4_)

As shown in figure 1(b), Fe_3_O_4_ has the face centered cubic spinel structure, based on 32 O^2−^ ions and close-packed along the [111] direction. Fe_3_O_4_ differs from most other iron oxides in that it contains both divalent and trivalent iron. Fe_3_O_4_ has a cubic inverse spinel structure that consists of a cubic close packed array of oxide ions, where all of the Fe^2+^ ions occupy half of the octahedral sites and the Fe^3+^ are split evenly across the remaining octahedral sites and the tetrahedral sites. In stoichiometric magnetite Fe^II^/Fe^III^ = 1/2, and the divalent irons may be partly or fully replaced by other divalent ions (Co, Mn, Zn, etc). Thus, Fe_3_O_4_ can be both an *n*- and *p*-type semiconductor. However, Fe_3_O_4_ has the lowest resistivity among iron oxides due to its small bandgap (0.1 eV) [12].

#### 1.1.3. Maghemite (*γ*-Fe_2_O_3_)

As shown in figure 1(c), the structure of *γ*-Fe_2_O_3_ is cubic; each unit of maghemite contains 32 O^2−^ ions, 21⅓ Fe^3+^ ions and 2⅓ vacancies. Oxygen anions give rise to a cubic close-packed array while ferric ions are distributed over tetrahedral sites (eight Fe ions per unit cell) and octahedral sites (the remaining Fe ions and vacancies). Therefore, the maghemite can be considered as fully oxidized magnetite, and it is an *n*-type semiconductor with a bandgap of 2.0 eV.

**Figure 2.**
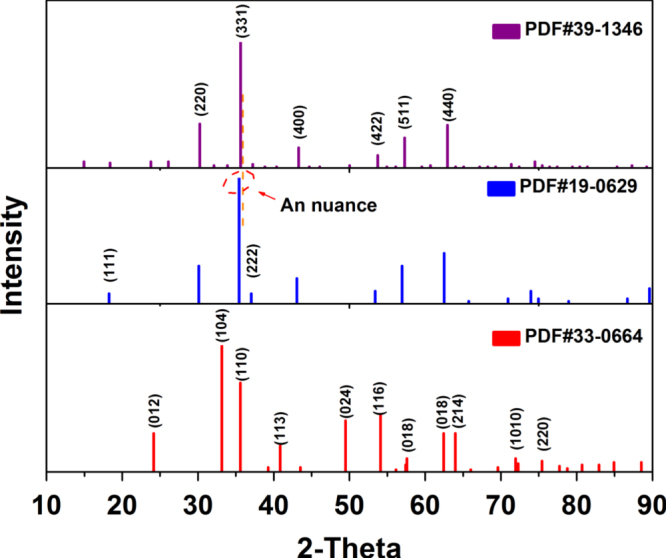
The XRD peak lines from standard powder diffraction files of *α*-Fe_2_O_3_ (33–0664), Fe_3_O_4_ (19–0629) and *γ*-Fe_2_O_3_ (39–1346).

Figure 2 shows the x-ray diffraction (XRD) peak lines from the standard powder diffraction files of *α*-Fe_2_O_3_ (33–0664), Fe_3_O_4_ (19–0629) and *γ*-Fe_2_O_3_ (39–1346), and it can be found that *γ*-Fe_2_O_3_ has a crystal structure similar to that of Fe_3_O_4_. The diffractogram of the cubic form of *γ*-Fe_2_O_3_ is identical to that of Fe_3_O_4_ with some line shift towards higher angles. It is noteworthy that the annealing treatment is a key step in most synthesis of different crystalline phase iron oxides. Any type of iron oxide can be obtained from the other types by oxidizing or reducing the annealing treatment. Thus, the XRD patterns are a basic characterization technique for determining the crystal structure and types of magnetic IONPs.

### 1.2. Size, shape and magnetic properties

Understanding the correlation between the magnetic properties and the size and shape of IONPs is a prerequisite for widespread applications of magnetism in data storage and bio-separation areas [13]. Generally, *α*-Fe_2_O_3_ has weak ferromagnetism at room temperature, while the saturation magnetization is often smaller than 1 emu g^−1^. However, *γ*-Fe_2_O_3_ and Fe_3_O_4_ exhibit ferrimagnetism at room temperature, with the saturation magnetization reaching to 92 emu g^−1^ [14]. It is noteworthy that many properties of IONPs depend on their size and shape. For example, Levy *et al* studied the magnetic properties of IONPs from 6 to 18 nm, the results revealed that magnetic disorder was particularly evident for 13–18 nm IONPs due to a drastic loss of their hyperthermia performance [15]. Guardia *et al* reported that pseudospherical and faceted IONPs with a narrow size distribution (4–20 nm) and a high saturation magnetization (*M*_s_ ≈ 80–85 emu g^−1^ at 5 K) were obtained by thermal decomposition using oleic acid as a surfactant. In contrast, decanoic acid yields much larger pseudocubic IONPs (45 nm) with a broader size distribution and a larger saturation magnetization (*M*_s_ = 92 emu g^−1^ at 5 K), which is close to the expected value for bulk magnetite [16]. One-dimensional iron oxide nanostructures are very appealing, owing to their many unique physicochemical properties based on high intrinsic anisotropy and surface activity. Recently, we showed a comparative study of the magnetic behavior of single and tubular clustered Fe_3_O_4_ NPs. The results revealed that the competition of the demagnetization energy of shape and the magnetocrystalline anisotropy energy of small IONPs would increase the coercivity, and the magnetic properties are strongly influenced by the morphology of the Fe_3_O_4_ NPs [17]. In general, IONPs become superparamagnetic at room temperature when the size of IONPs is below about 15 nm, meaning that the thermal energy can overcome the anisotropy energy barrier of a single nanoparticle. However, aggregation among superparamagnetic IONPs is a common phenomenon. Hence, for protecting bare IONPs against aggregation, the magnetic properties can be tailored by the coating materials, such as Au, Ag and Co_3_O_4_.

**Figure 3.**
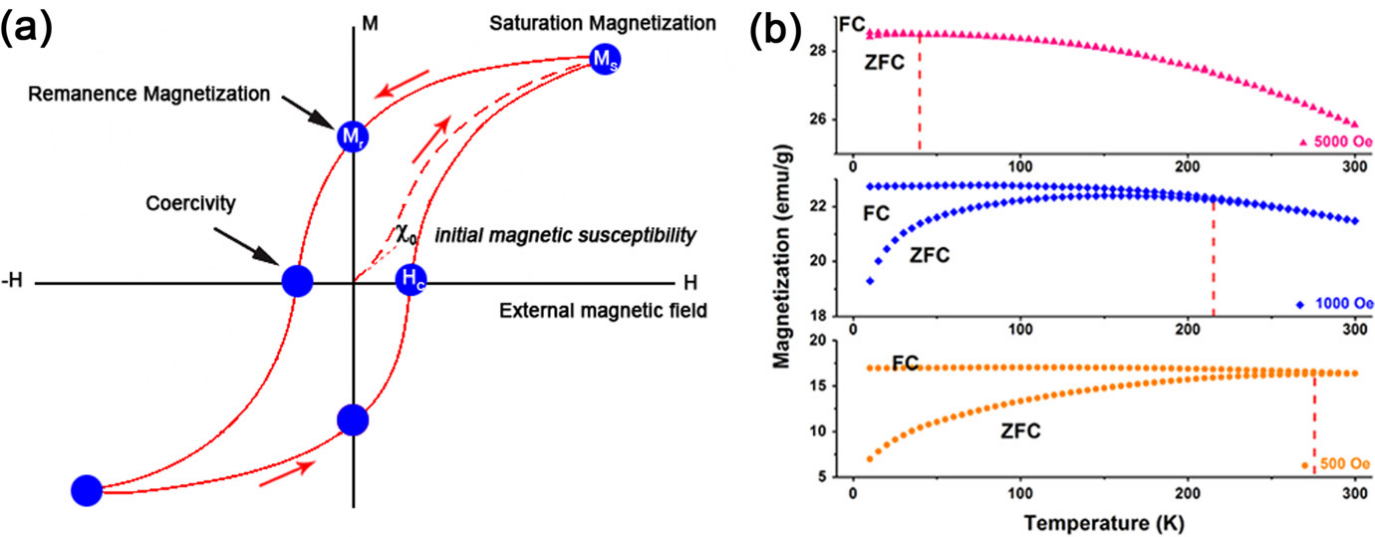
Schematic presentation of the typical hysteresis loops of IONPs (a); the ZFC/FC curves of *γ*-Fe_2_O_3_ at the different applied field (b).

There are a number of magnetic properties for characterization of IONPs. The most decisive properties are the response type to the magnetic field (including ferromagnetic, paramagnetic, antiferromagnetic and ferrimagnetic) and magnetization, which can be measured from the hysteresis loops (M–H) and zero-field cooled/field cooled (ZFC/FC, M–T) curves. As shown in figure 3(a), the saturation magnetization (*M*_s_), remanence magnetization (*M*_r_) and coercivity (*H*_C_) can be obtained from the hysteresis loops. When the IONPs are superparamagnetic, the M–H curve should show no hysteresis, and the forward and backward magnetization curves overlap completely and are almost negligible [17, 18]. As shown in figure 3(b), in ZFC measurements, the samples were cooled from 300 to 10 K without applying an external field. After reaching 10 K, an external field was applied, and the magnetic moments were recorded as the increased temperature. Conversely, for FC measurements, the samples were cooled from 300 K under an applied external field, and then the magnetic moments were recorded as the increased temperature. When the IONPs are cooled to the zero magnetic field temperature, the total magnetization of the IONPs will be zero since the magnetization of the individual IONPs is randomly oriented. An external magnetic field energetically favors the reorientation of the moments of the individual particle along the applied field at low temperatures. Thus, upon increasing the temperature, all ZFC magnetic moments increase and reach a maximum, where the temperature is referred to as the blocking temperature (*T*_B_). *T*_B_ is defined as the temperature at which NPs’ moments do not relax (known as blocked) during the time scale of the measurement [19, 20]. The high field can lower the energy barriers between the two easy axis orientations, therefore, lowering the blocking temperature. Moreover, if the applied field reaches a critical value, the blocking temperature will disappear [13].

## 2. Synthesis methods of magnetic IONPs

To date, a variety of synthetic methods such as co-precipitation, thermal decomposition, hydrothermal and solvothermal syntheses, sol–gel synthesis, microemulsion, ultrasound irradiation and biological synthesis have been applied to produce magnetic IONPs. These methods can be divided into aqueous and non-aqueous routes. Aqueous approaches are attractive in terms of their low cost and sustainability; there is, however, a generic challenge in directly obtaining water-soluble monodisperse magnetic IONPs without size selection. Non-aqueous routes generally obtained IONPs which only dissolved in nonpolar solvents. Various magnetic nanostructures with different morphologies have been synthesized, including particles, wires, and rods.

### 2.1. Co-precipitation

As the most conventional method, the co-precipitation method consists of mixing ferric and ferrous ions in a 1:2 molar ratio in very basic solutions at room temperature or at elevated temperature. The reaction mechanism can be simplified as: Fe^2+^ + 2Fe^3+^ + 8OH^−^ ⇆ Fe(OH)_2_ + 2Fe(OH)_3_ → Fe_3_O_4_↓ + 4H_2_O. Usually, the reaction undergoes gas protection. The nucleation of the Fe_3_O_4_ nucleus is easier when the solution pH is lower than 11, while the growth of the Fe_3_O_4_ nucleus is easier when the solution pH is higher than 11.

After the pioneering work prepared by Massart [21], co-precipitation was widely studied in preparing Fe_3_O_4_ NPs for its extraordinary advantages, such as gram-scale production and facility. We have reported the large-scale co-precipitation synthesis of Fe_3_O_4_ NPs, where their corresponding morphology, structure, and magnetic properties at different reaction temperatures were investigated [22]. Recently, several modified co-precipitation methods have been developed; for example, as reported by Wu *et al*, magnetic Fe_3_O_4_ nanopowders with an average diameter of 15 nm were synthesized by ultrasonic-assisted chemical co-precipitation utilizing high purity iron separated from iron ore tailings by an acidic leaching method [23]. The present synthesis method of Fe_3_O_4_ NPs easily yields SPIONPs without a protecting gas. Recently, superparamagnetic Fe_3_O_4_ NPs with sizes of 4.9–6.3 nm were synthesized by a one-step aqueous co-precipitation route based on the use of alkanolamines as the base, the reported methodology provides a simple, versatile, and cost-effective route for the high-yield synthesis of IONPs featuring improved magnetic properties and small particle sizes [24]. Typically, small size leads to low magnetic properties; the above results showed improved magnetic properties while keeping their small size.

**Figure 4.**
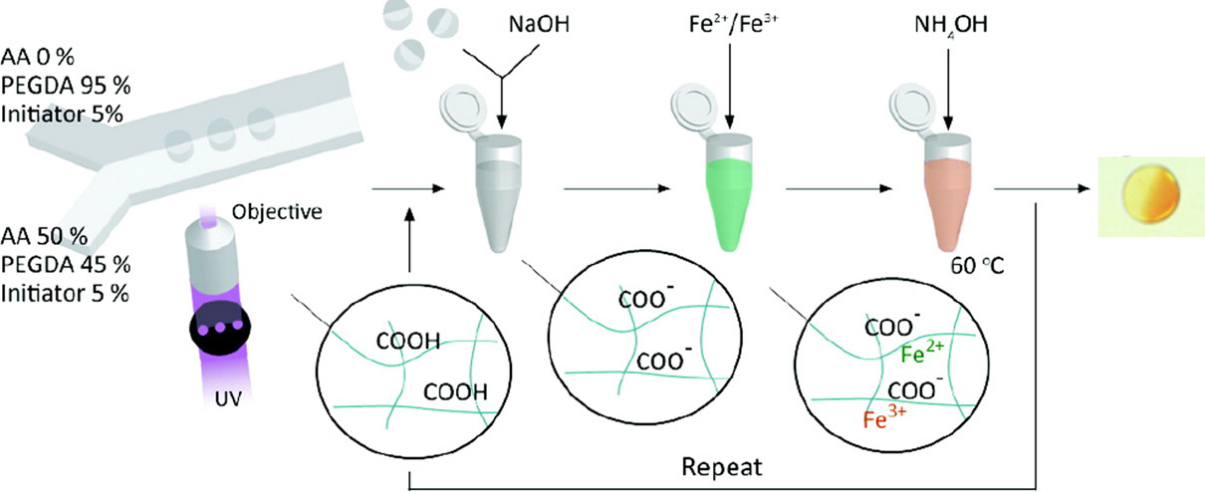
Schematic showing the *in situ* co-precipitation synthesis process of IONPs in polymer. (Reprinted with permission from S K Suh *et al* 2012 *J. Am. Chem. Soc.* **134** 7337. Copyright 2012 American Chemical Society.)

Currently, the problems of aggregation and biocompatibility of IONPs perhaps hinder the applications in biomedical fields. Therefore, many surfactants and biomolecules have been introduced directly in the co-precipitation process. For instance, Salavati-Niasari *et al* have reported Fe_3_O_4_ NPs with a size range of 25 nm that were prepared by a facile chemical co-precipitation method; the surfactant octanoic acid was present in the reaction system to improve the dispersity [25]. Liu *et al* have prepared magnetic chitosan coated Fe_3_O_4_ NPs by the co-precipitation method under 0.45 T static magnetic fields, which assisted the glutaraldehyde cross-linking reaction; the water was replaced by 2% chitosan in an acetic acid solution during the reaction process. The resulting NPs were used to immobilize lipase [26]. Recently, Suh *et al* have reported an *in situ* synthesis of nonspherical magnetic IONPs in a carboxyl functionalized polymer matrix, in which the iron ions diffused into the polymer particles and they were allowed to chelate with the deprotonated carboxyl groups, nucleated and finally grew to the IONPs in the polymer particles (figure 4) [27]. This method can be used to add multiple functionalities, such as the addition of biomolecules after subsequent reactions.

However, control over particle size, morphology and composition in the co-precipitation route is limited as particle kinetically controlled growth. The size, shape and composition of the IONPs depend on the experimental parameters, such as the types of iron salts (chlorides, perchlorates, sulfates, nitrates, etc), Fe(II)/Fe(III) ratio, pH value and ionic strength of the medium. For example, Blanco-Andujar *et al* have synthesized uncoated IONPs by using sodium carbonate as a co-precipitating agent; the reaction proceeded sufficiently slowly to enable a detailed study of both the reaction pathway and products [28]. Pereira *et al* have synthesized superparamagnetic ferrite NPs (MFe_2_O_4_, where M = Fe, Co, Mn) through a novel one-step aqueous co-precipitation method on the basis of using a new type of alkaline agent, including alkanolamines isopropanolamine and diisopropanolamine. Remarkably, the resulting NPs exhibited smaller particle sizes (up to 6 times) and enhanced saturation magnetization (up to 1.3 times) relative to those prepared with NaOH [24]. We also investigated the effect of the drying method on the change of the morphology and magnetic properties of IONPs, and the results revealed that NPs obtained by vacuum drying tend to be agglomerated more easily when the average diameter of the grain decreased in pace with the evaporation of the surface adsorptive water and inner containing water, though the structure and morphology are maintained better by ambient air drying. Among all the drying treatments, the highest saturation magnetization was obtained after drying in a vacuum at 70 °C. This finding is instructive to elucidate in depth some relationship between structure and magnetic property [22].

Although the co-precipitation method is one of the successful and classical techniques for synthesizing IONPs with high saturation magnetization, more attention should be paid to overcoming the shortcomings of this method, such as the broad particle size distribution of products, and the utilization of a strong base in the reaction process.

### 2.2. High-temperature thermal decomposition

The above co-precipitation method involves fast particle formation rates and therefore, particle size and size distribution are hardly controlled. To avoid the limitations of this method, different alternative strategies have been developed, such as nonaqueous thermal decomposition strategies. In principle, the thermal decomposition strategies can be subdivided into hot-injection approaches, where the precursors are injected into a hot reaction mixture, and conventional reaction strategies where a reaction mixture is prepared at room temperature and then heated in a closed or open reaction vessel.

Due to the fact that most of the reactions are carried out at room temperature in co-precipitation, the obtained IONPs often exhibit low crystallinity. In contrast, higher monodisperse, narrow size distribution and highly crystalline magnetic IONPs are obtained from high-temperature thermal decomposition of organometallic or coordinated iron precursors in organic solvents, which display superior properties to those obtained by co-precipitation, since nucleation can be separated from growth and complex hydrolysis reactions can be avoided [29, 30]. The as-used ferric salts include Fe(CO)_5_ [31], Fe(acac)_3_ (acac = acetylacetonate) [32], iron oleate [33], Fe(Cup)_3_ (Cup = N-nitrosophenylhydroxylamine) [34, 35], Prussian blue (Fe_4_[Fe(CN)_6_·14H_2_O] [36, 37], Fe–urea complex ([Fe(CON_2_H_4_)_6_](NO_3_)_3_) [38], ferrocene (Fe(C_5_H_5_)_2_) [39], and Fe_3_(CO)_12_ [40]. To obtain monodisperse IONPs, various organic molecules including oleic acid, 1-octadecene, 1-tetradecene, and oleylamine, are often added in the reaction process as stabilizers. The stabilizer can slow down the nucleation process and it affects the adsorption of additives on the nuclei and the growing nanocrystals, which may inhibit the growth of the IONPs, and favor the formation of small IONPs. The as-obtained products are usually spherical NPs with sizes of below 30 nm and their size-distribution can only be controlled to a small extent.

**Figure 5.**
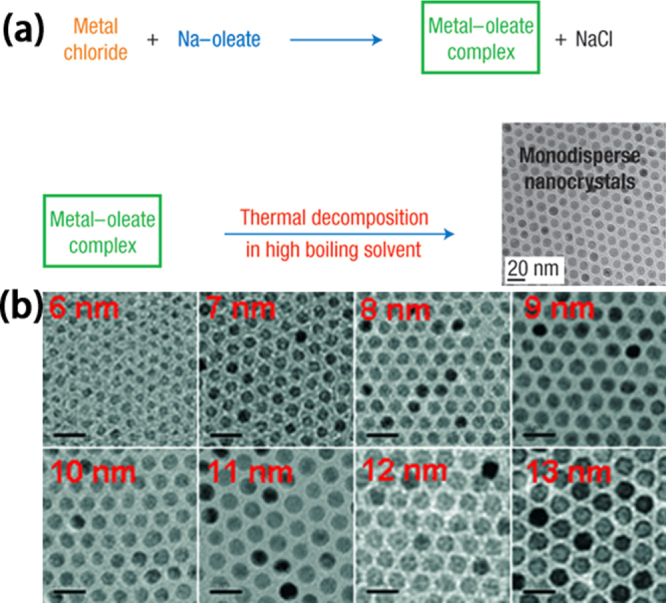
Metal–oleate precursors were prepared from the reaction of metal chlorides and sodium oleate. The thermal decomposition of the metal–oleate precursors in the high boiling solvent produced monodisperse nanocrystals (a). (Reprinted with permission from J Park *et al* 2004 *Nat. Mater.* **3** 891. Copyright 2004 Nature Publishing Group.) Transmission electron microscopy (TEM) images of 6, 7, 8, 9, 10, 11, 12, and 13 nm-sized air-oxidized IONPs showing the one nanometer level increments in diameter (b). (Reprinted with permission from J Park *et al* 2005 *Angew. Chem. Int. Edn* **44** 2872. Copyright 2005 John Wiley and Sons.)

As shown in figure 5, Hyeon *et al* have reported a synthetic method of obtaining monodisperse IONPs by using inexpensive and nontoxic iron chloride rather than toxic and expensive iron pentacarbonyl. An organic solvent dispersion containing the iron–oleate complex and a surfactant was slowly heated to the boiling point of the solvent to produce monodisperse IONPs. In a single reaction, as much as 40 g of monodisperse IONPs was generated without any size-selection process [41]. The size of the IONPs was controlled by changing the aging temperature and other parameters. This concept of continuous growth without additional nucleation could be applicable to other materials, and the synthetic procedure is highly reproducible.

**Figure 6.**
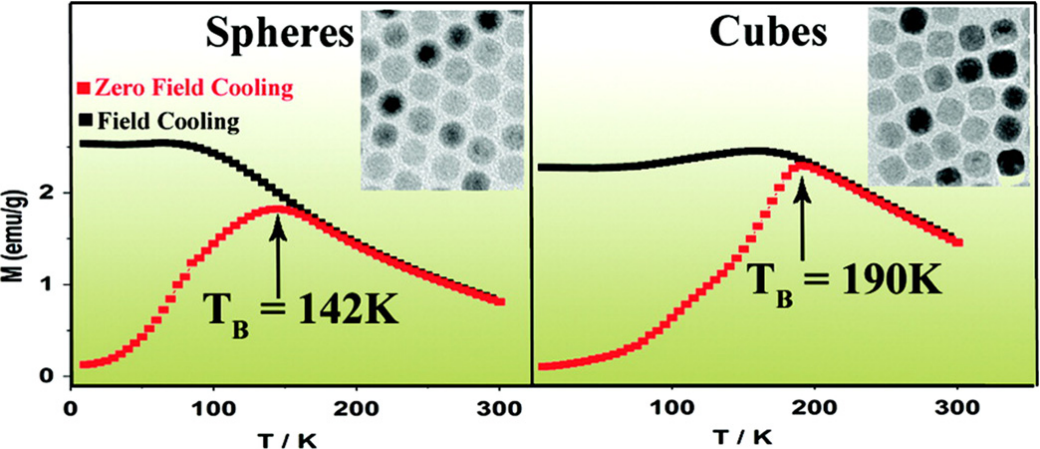
Monodisperse IONPs with spherical and cubic morphologies are prepared by the thermal decomposition of FeOOH, and exhibit very different blocking temperatures. (Reprinted with permission from R Chalasani and S Vasudevan 2011 *J. Phys. Chem.* C **115** 18088. Copyright 2010 American Chemical Society.)

Moreover, the thermal decomposition method is often used to prepare iron oxide with different shapes, such as nanocubes and nanospheres. For example, Amara *et al* synthesized Fe_3_O_4_ nanocubes and nanospheres by solventless thermal decomposition of various mixtures of ferrocene and polyvinylpyrrolidone (PVP). The described method offered a new simple, single-step process for the preparation of magnetite nanocubes/spheres [42]. As shown in figure 6, Chalasani and Vasudevan have reported monodisperse iron oxide nanocrystals with spherical and cubic morphologies that were prepared by the thermal decomposition of FeOOH. The higher *T*_B_ values in particles of cubic morphology are shown to be a consequence of exchange bias fields. The results reveal that the exchange bias fields originate from the presence of trace amounts of wustite, FeO [43]. In fact, the magnetic properties of IONPs are also associated with the shape and size of the NPs [16, 44, 45].

Additionally, the shape and size of IONPs can also be tailored by the use of different precursors, additives and solvents during the thermal decomposition process. Shavel and Liz-Marzán have reported a detailed overview on the effect of various synthesis parameters during the synthesis process of IONPs with different shapes, through high-temperature decomposition of a preformed iron oleate complex. While this procedure has been previously shown to produce monodisperse magnetite spheres, the use of specific additives is demonstrated to allow for the preparation of strongly faceted iron oxide nanocrystals, with either cubic or octahedral shapes. Additionally, using squalene or octadecene as the solvent was found to induce the reduction of the iron precursors and thereby lead to the formation of NPs with core–shell (in the case of nanocubes) or island-like structures (in the case of octahedrons) of Fe^0^/iron oxide [46]. Demortière *et al* reported a fine control of IONP diameters from 2.5 to 14 nm by using different types of solvents, including eicosene (14 nm), di-*n*-octyl ether (11 nm), dibenzyl ether (9 nm), di-*n*-octyl ether (5 nm), hexadecene (3.5 nm), and di-*n*-hexyl ether (2.5 nm) [47].

Hyeon’s method is commonly used for the synthesis of monodisperse and reproducible IONPs with a tailored size [41, 48]. However, the nucleation of IONPs in thermal decomposition involves boiling the solvents, so the accurate shape of the IONPs is not fully reproducible. Recently, Lynch *et al* conducted a mechanistic study on the synthesis of colloidal IONPs by thermal decomposition; gas bubbles were generated by boiling solvents or artificial Ar bubbling, and the results illustrated that gas bubbles had a stronger effect on the nucleation of IONPs than on their growth [49]. It is noteworthy that the IONPs resulting from the thermal decomposition method are usually dissolved in nonpolar solvents.

### 2.3. Hydrothermal and solvothermal synthesis

The aqueous solution route is used for the fabrication of *α*-Fe_2_O_3_ and Fe_3_O_4_ NPs; the solution synthesis for *γ*-Fe_2_O_3_ usually involved the controlled oxidation of Fe_3_O_4_ and the direct mineralization of Fe^3+^ ions. Subsequently, other non-aqueous solution methods have also been developed to synthesize highly crystalline, monodisperse, and shape-controlled *γ*-Fe_2_O_3_ NPs, in which organometallic compounds were always used as precursors. However, hydrothermal or solvothermal synthesis includes various wet-chemical techniques of crystallizing the substance in a sealed container from the high temperature aqueous or non-aqueous solution (generally in the range 130–250 °C) under high vapor pressure (generally in the range 0.3–4 MPa) [1]. This method has also been used to grow dislocation-free single crystal particles, and grains formed in this process could have a better crystallinity than those from other processes, and hence hydrothermal and solvothermal synthesis are prone to obtaining highly crystalline IONPs, including *α*-Fe_2_O_3_, *γ*-Fe_2_O_3_, and Fe_3_O_4_ NPs.

Possible advantages of the hydrothermal method over other types of crystal growth include the ability to create crystalline phases which are not stable at the melting point. In addition, materials which have a high vapor pressure near their melting points can also be grown by the hydrothermal method [50]. The method is also particularly suitable for the growth of good-quality iron oxide nanocrystals while maintaining good control over their composition. It has to be pointed out that the concepts embodied in the hydrothermal process have already been extrapolated to non-aqueous systems, and the so-called ‘solvothermal process’ has emerged, in which an organic solvent is used as the reaction medium instead of water. See [51] for an overview of the current state-of-the-art of hydrothermal synthesis routes for the synthesis of a rich family of IONPs with different shapes or assembled complex nanostructures.

Moreover, the hydrothermal and solvothermal route is a facile and conventional method for obtaining hollow IONPs. In a typical procedure, using Fe^3+^ as the iron resource, acetate, urea, and sodium citrate are mixed in ethylene glycol under stirring, then the resultant homogeneous dispersion is transferred to a Teflon-lined stainless steel autoclave and sealed to heat at about 200 °C for 8–24 h [52–55]. Furthermore, the hydrothermal and solvothermal synthesis route has been developed to prepare IONPs with controllable size and shape [56–59]. For example, Ma *et al* developed a facile template-free synthetic route for the controlled fabrication of various *α*-Fe_2_O_3_ nanostructures, such as small NPs, nanopolyhedra, and NP-aggregated microcubes, by simply controlling the synthesis parameters such as reaction time and solvent [60]. Recently, Tian *et al* reported a facile solvothermal approach to synthesize ultrasmall monodisperse Fe_3_O_4_ NPs with a precise size control of 1 nm, in which Fe(acac)_3_ acted as an iron source, *n*-octylamine as a reductant, and *n*-octanol as a solvent [61].

**Figure 7.**
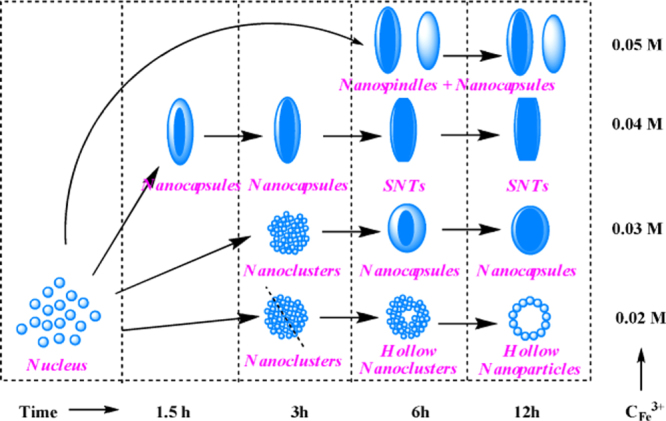
Schematic illustration of the shape evolution for hematite nanostructures at different reaction times and different ferric concentrations. (Reprinted with permission from W Wu *et al* 2010 *J. Phys. Chem.* C **114** 16092. Copyright 2010 American Chemical Society.)

In addition, the hydrothermal and solvothermal route is beneficial to obtaining shape-controlled IONPs. We present a facile approach for the production of magnetic iron oxide short nanotubes (SNTs) and other shapes (NPs, nanorings) employing an anion-assisted hydrothermal route by using phosphate and sulfate ions. As shown in figure 7, the size, morphology, shape, and surface architecture control of the iron oxide SNTs are achieved by simple adjustments of ferric ion concentration without any surfactant assistance. Investigation of the formation mechanism reveals that the ferric ion concentrations, the amount of anion additive, and the reaction time contribute significantly to SNT growth. The shape of the SNTs is mainly regulated by the adsorption of phosphate ions on faces parallel to the long dimension of elongated *α*-Fe_2_O_3_ NPs (axis) during nanocrystal growth, and the hollow structure is given by the preferential dissolution along the *c*-axis due to the strong coordination of the sulfate ions. Moreover, the as-synthesized hematite (*α*-Fe_2_O_3_) SNTs can be converted to magnetite (Fe_3_O_4_) and maghemite (*γ*-Fe_2_O_3_) ferromagnetic SNTs by a reducing atmosphere annealing process while preserving the same morphology [10].

### 2.4. Sol–gel reactions and polyol method

The sol–gel process is a classical wet-chemical technique widely used in the fields of materials science and ceramic engineering. Such a method is used primarily for the fabrication of materials (typically metal oxides). Generally, it involves starting from a colloidal solution that acts as the precursor for an integrated network of either discrete particles or network polymers. In this system, a sol is a stable dispersion of colloidal particles or polymers in a solvent. A gel consists of a three dimensional continuous network, which encloses a liquid phase. In a colloidal gel, the network is built from the agglomeration of colloidal particles. In a polymer gel, the particles have a polymeric sub-structure made by aggregation of sub-colloidal particles. Generally, sol particles may interact by Van der Waals forces or hydrogen bonds, and a gel may also form from linking polymer chains. In most gel systems used for materials synthesis, the interactions are of a covalent nature and the gel process is irreversible. The gelation process may be reversible if other interactions are involved. Typical precursors for the synthesis of IONPs are iron alkoxides and iron salts (such as chlorides, nitrates and acetates), which undergo various forms of hydrolysis and polycondensation reactions [62]. These reactions are performed at room temperature, and further heat treatments are needed to acquire the final crystalline state. By this method, the IONPs will form through at least a two-step phase transformation: Fe(OH)_3_ → *β*-FeOOH → *γ*-Fe_2_O_3_ [63]. The final properties of IONPs are highly dependent upon the structure created during the sol stage of the sol–gel process. For example, Lemine *et al* reported Fe_3_O_4_ NPs with an average particle size of 8 nm were successfully prepared by the sol–gel method. The saturated magnetization could be up to 47 emu g^−1^ at room temperature, and it was expected that these NPs were promising materials for biomedical applications [64]. Recently, Qi *et al* reported Fe_3_O_4_ NPs in the interval of 9 ∼ 12 nm that were synthesized by a non-alkoxide sol–gel method. Through this technique, sol–gel materials were prepared from ethanolic solutions of metal chlorides without the need for alkoxides, polymeric gel agents, or elaborate reaction schemes [65].

The different organic precursors are the crucial roles in controlling the shape and crystal structure of IONPs. For example, Woo *et al* have described a sol–gel mediated synthesis of Fe_2_O_3_ nanorods with controlled phase depending on the conditions. The diameter and length of the nanorods could be controlled by the H_2_O/oleic-acid ratio in the gelation process, and the phase of the nanorods could be controlled by the temperature, atmosphere, and hydrous state of the gels during crystallization [66]. In our previous report, water-soluble hollow spherical Fe_3_O_4_ nanocages (about 100 nm) with high saturation magnetization were prepared by applying glutamic acid as an additive in a one-pot sol–gel process and subsequent annealing to synthesize *γ*-Fe_2_O_3_ nanocages with similar nanostructures. The results indicated that glutamic acid played an important role in the formation of the cage-like nanostructures [18, 67].

The polyol method is also understood as an inversed sol–gel method (the sol–gel method uses an oxidation reaction but polyol synthesis uses a reduction reaction), which is well suited for the preparation of IONPs with various shapes and sizes [68]. In the polyol synthesis process, the polyols not only serve as solvents but also reducing agents, which apply as stabilizers to control particle growth and prevent interparticle aggregation. In the typical reaction process, an iron precursor compound is suspended in a liquid polyol. The suspension is stirred and heated to a given temperature that can reach the boiling point of the polyol. Compared to the hydrothermal method, this reaction does not require high pressure, thus, it is unnecessary to operate in the Teflon-lined stainless steel autoclave. For example, Cai and Wan fabricated monodisperse Fe_3_O_4_ NPs by utilizing four types of polyols to reduce Fe(acac)_3_ in a similar reaction procedure, including ethylene glycol (EG), diethylene glycol (DEG), triethylene glycol (TREG) and tetraethylene glycol (TEG). Only the TREG can yield non-agglomerated Fe_3_O_4_ NPs with uniform shape and narrow size distribution. The result illustrates that the polyol solvent plays a crucial role in determining the morphology and colloidal stability of the resulting particles [69]. Indeed, the polyol solvent also plays an important role in determining the size and magnetic properties of IONPs: different polyols will generate IONPs with different sizes [70, 71].

In comparison with the co-precipitation method, the sol–gel and polyol methods for IONPs have several advantages. For example, the IONPs can be easily dispersed in aqueous media and other polar solvents because the surface of IONPs contain many hydrophilic ligands. Also the relatively high reaction temperature of these two methods favors IONPs with higher crystallinity and saturation magnetization. Nevertheless, the disadvantages of the sol–gel process are the relatively high cost of the metal alkoxides and the release of large amounts of alcohol during the calcination step, requiring safety considerations during the sol–gel process.

### 2.5. Microemulsion

Microemulsions are clear, stable, and isotropic liquid mixtures of oil, water and surfactant, frequently in combination with a co-surfactant. The surfactant molecules may form a monolayer at the interface between the oil and water, with the hydrophobic tails of the surfactant molecules dissolved in the oil phase and the hydrophilic head groups in the aqueous phase, and vice versa. In this system, the aqueous phase may contain metal salts and/or other ingredients, and the ‘oil’ may actually be a complex mixture of different hydrocarbons and olefins. The two basic types of microemulsions are direct (oil dispersed in water, o/w) and reversed (water dispersed in oil, w/o), which have all been used to synthesize IONPs with tailored shape and size. Common surfactants including bis(2-ethylhexyl) sulfosuccinate (AOT), sodium dodecyl sulfate (SDS), cetyltrimethylammonium bromide (CTAB), and PVP have been widely used in the fabrication of magnetic IONPs [72–74]. Generally, the size control and the dynamics of IONP formation can be achieved by varying, for instance, the droplet size, the initial concentration of reactants and the nature of surfactants.

Recently, Darbandi *et al* reported that uniformly sized and crystalline IONPs with a spinel structure and mean diameters of about 3, 6 and 9 nm were synthesized in high yield using the microemulsion route at room temperature. During this process, the capping agent (polyoxyethylene (5) nonylphenylether as surfactant) was capable of preventing the agglomeration effect, which can occur in case of direct particle contact [75]. Okoli *et al* synthesized magnetic IONPs for protein binding and separation using w/o and o/w microemulsions, respectively. The potential of both approaches for the production of nanocrystalline magnetic IONPs with high surface area for protein binding/protein purification are investigated and compared. The average specific surface areas of the IONPs are 147 m^2^ g^−1^ for w/o and 304 m^2^ g^−1^ for o/w microemulsions. A higher specific surface area seen in o/w microemulsions is attributed to the small size of the nanoparticle. The protein bound IONPs exhibited a significant reduction of the removal rate of clay particles in suspension as compared to bare IONPs, evidencing a significant interaction between the magnetic IONPs and the protein [76, 77].

However, despite the presence of surfactants, the aggregation of the produced magnetic IONPs usually requires several washing processes and further stabilization treatments for them to be used in biomedical applications.

### 2.6. Sonolysis or sonochemical method

The sonolysis (sonochemical or ultrasound irradiation) method uses the chemical effects of ultrasound arising from acoustic cavitation. High intensity ultrasound is used for the production of novel structures and provides an unusual route to known materials without bulk high temperatures, high pressures, or long reaction times [78]. Under ultrasound irradiation, the alternating expansive and compressive acoustic waves create bubbles (i.e., cavities) and make the bubbles oscillate. The oscillating bubbles can accumulate ultrasonic energy effectively while growing to a certain size (typically tens of mm). Under the right conditions, a bubble can overgrow and subsequently collapse, releasing the concentrated energy stored in the bubble within a very short time (with a heating and cooling rate of >10^10^ K s^−1^). This cavitational implosion is really localized and transient with a temperature of 5000 K and a pressure of 1000 bar [1, 79, 80]. Therefore, the sonolysis method is employed to prepare various forms of bare and functionalized IONPs by the sonication of an aqueous ferro or ferrous salt solution, and the experimental process is often carried out under ambient conditions (usually in the presence of air) [81–85].

The sonolysis method can be used to synthesize biocompatible IONPs. For instance, Theerdhala *et al* reported on the binding of a semi-essential amino acid, L-arginine, onto the surface of Fe_3_O_4_ NPs, creating a stable aqueous suspension by a one-step method through sonochemical synthesis. These surface-functionalized IONPs could become a promising vehicle for drug delivery [86]. Recently, Zhu *et al* synthesized Fe_3_O_4_ NPs of 30–40 nm by a sonochemical method, and these NPs were uniformly dispersed on reduced graphene oxide sheets (Fe_3_O_4_/RGO). The composite Fe_3_O_4_/RGO was immobilized with hemoglobin to fabricate a biosensor for detecting H_2_O_2_. The biosensor demonstrated a fast response to H_2_O_2_ (within 10 s) and displayed an excellent linear relationship at 4 × 10^−6^ to 1 × 10^−3^ M with the detection limit of 2 × 10^−6^ M (S/N = 3) [87].

**Figure 8.**
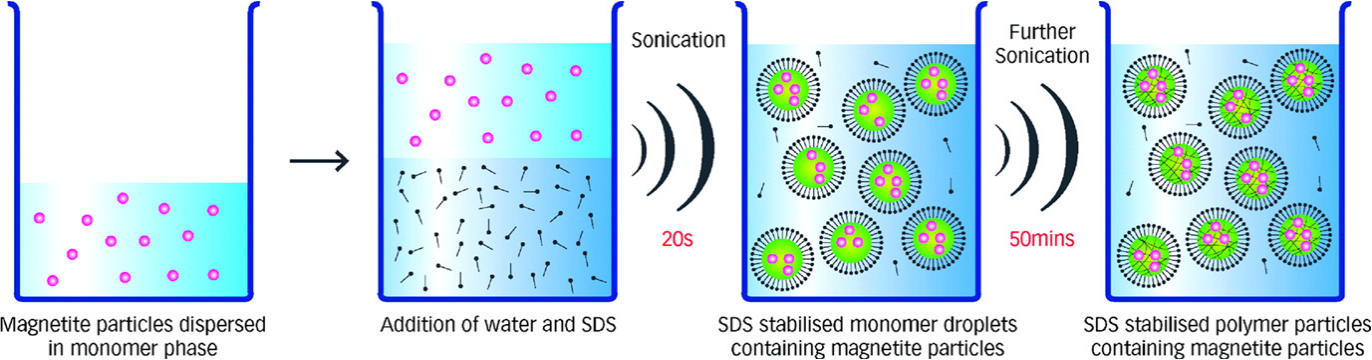
Schematic diagram of the procedure for the encapsulation of Fe_3_O_4_ NPs and monomer droplet to latex particle conversion by the sonochemically driven miniemulsion polymerization pathway. (Reprinted with permission from B M Teo *et al* 2009 *Langmuir* **25** 2593. Copyright 2009 American Chemical Society.)

In addition, the ultrasound-initiated procedure, as a technology, represents an effective and innocuous means of producing a range of nanocomposites, consisting of multiple combinations of different polymers and encapsulated materials. Teo *et al* have developed a simple and efficient method for preparing 100 nm latex beads loaded with a high content of Fe_3_O_4_ NPs; the formation procedure of Fe_3_O_4_ NPs under the ultrasound-initiated effect is well illustrated in figure 8. The NPs exhibited excellent colloidal stability (remained suspended stably in an aqueous solution for more than 12 months with no noticeable degradation) and strong magnetic properties (superparamagnetic with a saturated magnetization of 24 emu g^−1^), and were of the desired size to be technologically relevant [88]. The sonochemical method has some advantages, including uniformity of mixing and reduction of crystal growth, which can also lead to an acceleration effect in chemical dynamics and rates of the reactions. However, the sonolysis method is not beneficial to realize the fabrication of IONPs with controllable shapes and dispersity.

### 2.7. Microwave-assisted synthesis

It has long been known that molecules undergo excitation with electromagnetic radiation. This effect is utilized in household microwave ovens to heat food. However, microwave-assisted synthesis has only been used as a reaction methodology by chemists for a few years. Excitation with microwave radiation results in the molecules aligning their dipoles within the external field. Strong agitation, provided by the reorientation of molecules, in phase with the electrical field excitation, causes an intense internal heating. Therefore, microwave-assisted synthesis can significantly reduce the processing time and energy cost, due to its almost instantaneous ‘in core’ heating of materials in a homogeneous and selective manner, different from the classical ones.

**Figure 9.**
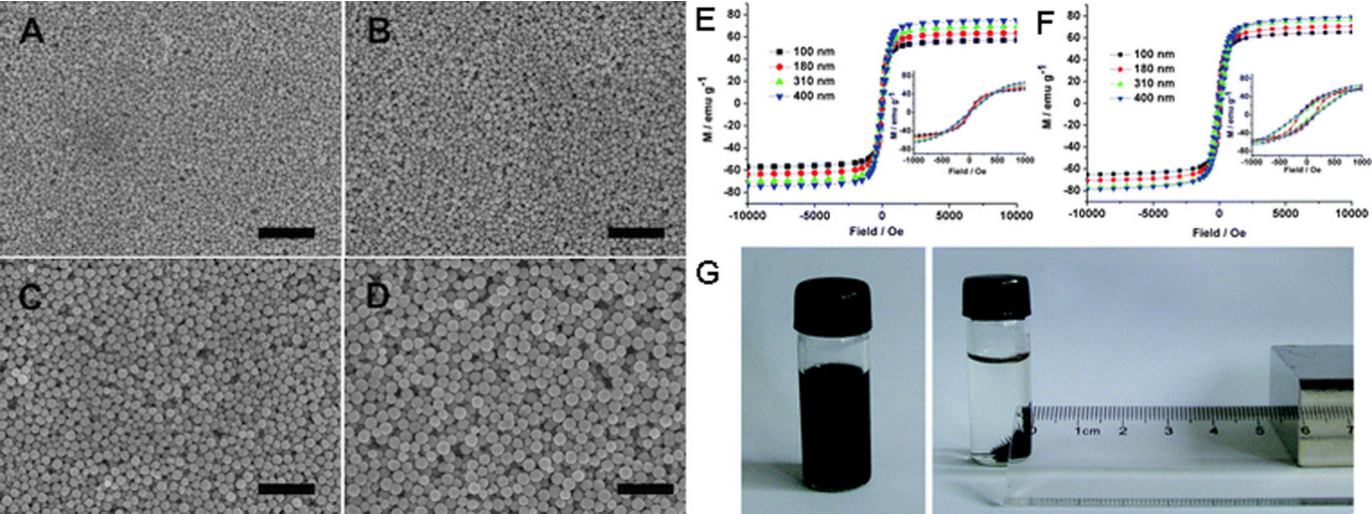
Typical SEM images of the polyacrylic acid-Fe_3_O_4_ hybrid nanostructure synthesized using different initial iron amounts of 0.7 mmol (A), 1.5 mmol (B), 3.0 mmol (C), and 5.0 mmol (D). All of the scale bars are 2 *μ*m. Magnetization curves of the hybrid nanostructure with different sizes at a temperature of 300 K and 1.8 K. Insets show the data around zero field with an expanded scale ranging from −1000 to 1000 Oe (E), (F). Photographs of a solution of the hybrid nanostructure with the diameter of 400 nm in the absence and presence of a magnet (G). (Reprinted with permission from S Liu *et al* 2011 *CrystEngComm* **13** 2425. Copyright 2011 Royal Society of Chemistry.)

The microwave-assisted synthesis method has been widely used to prepare magnetic IONPs with controllable size and shapes recently [89–92]. For example, Sreeja and Joy reported the fabrication of superparamagnetic *γ*-Fe_2_O_3_ NPs with an average diameter of 10 nm using the microwave-assisted method at 150 °C, in a short time-duration of 25 min. Their work showed that lower temperature and less reaction time were required to obtain comparable results by microwave heating [93]. Jiang *et al* have reported cubic IONPs that were prepared via the microwave-assisted method followed by Ostwald ripening procedures. The results illustrated the phase and magnetic properties of IONPs would change by varying the experimental conditions [94]. Indeed, the phase of IONPs by the microwave-assisted synthesis could be slightly different depending on the experimental conditions. For instance, Hu *et al* synthesized three major iron oxide phases: magnetite, maghemite and hematite, under microwave treatment in an autoclave, from alcohol/water solutions of chloride salts in the presence of NaOH. The results revealed that the pure hematite phase can be obtained in the presence of single precursor FeCl_3_. When FeCl_2_ was used as the single precursor, magnetite or maghemite NPs were produced depending on the drying process used [95]. Additionally, the microwave-assisted synthesis method is often employed to prepare biocompatible magnetic IONPs. Recently, Osborne reported a rapid and straightforward microwave-assisted synthesis of superparamagnetic dextran-coated IONPs. The NPs were produced in two hydrodynamic sizes with differing core morphologies by varying the synthetic process. The IONPs are found to be superparamagnetic and exhibit properties consistently in MRI. In addition, the dextran coating imparts the water solubility and biocompatibility necessary for *in vivo* utilization [96]. As shown in figure 9, Zhu *et al* reported polyacid-conjugated Fe_3_O_4_ superparamagnetic hybrid nanostructures that were conveniently fabricated by the introduction of a microwave-assisted method. The hybrid nanostructure was composed of superparamagnetic magnetite nanograins and presented a cluster-like structure; and its size range can be tuned from about 100–400 nm by varying the amount of FeCl_3_ in the system. The hybrid nanostructure exhibits excellent magnetic responsibility and good biocompatibility, which offers advantageous functionality due to the preferential exposure of uncoordinated carboxylate groups on its surface [97]. Compared to the thermal decomposition method, the stabilization of the IONPs prepared by the microwave-assisted synthesis route in organic solvents can be easily dispersed in water without laborious ligand exchange or purification steps. Such characteristics can be considered as attractive for fabrication of large-scale IONPs [98].

### 2.8. Biosynthesis

Biosynthesis of IONPs is a kind of bottom-up approach where the main reaction occurring is reduction/oxidation. The microbial enzymes or the plant phytochemicals with anti-oxidant or reducing properties are usually responsible for the reduction of salts into their respective NPs [99]. Generally, the biosynthesis method is a green chemical and eco-friendly route, and the obtained products exhibit a good biocompatibility. In the traditional biosynthesis for magnetic IONPs, magnetotactic bacteria and iron reducing bacteria are used, such as *Geobacter metallireducens*, *M*. *gryphiswaldense*, etc [100–102].

Recently, new types of bacteria have been employed to synthesize magnetic IONPs. For example, Bharde *et al* have reported that the bacterium *Actinobacter* sp. was capable of synthesizing maghemite NPs under aerobic conditions when reacted with a ferric chloride precursor. Moreover, maghemite NPs showed superparamagnetic characteristics as expected. Compared to the earlier reports of synthesis of magnetite NPs by magnetotactic bacteria and iron reducing bacteria, which took place strictly under anaerobic conditions, the present procedure offered a significant advance since the reaction occurred under aerobic conditions [103]. Recently, Sundaram *et al* reported the ability of *Bacillus subtilis* strains isolated from rhizosphere soil to produce IONPs. This successful synthesis of stabilized Fe_3_O_4_ NPs, which was capped by organic molecules, indicates the applicability of the isolated *Bacillus subtilis* strain for the bulk synthesis of IONPs [104].

Currently, how to control the size and shape of magnetic IONPs during biosynthesis processes, and the elucidation of the exact mechanism of IONPs production using living organisms, require much more experimentation.

### 2.9. Other methods

Except for the above-mentioned methods, numerous chemical or physical methods can also be used to synthesize magnetic IONPs, such as electrochemical methods [105–107], flow injection synthesis [108], and aerosol/vapor methods [109–111].

The electrochemical methods for IONPs present some advantages over other methods, the crucial one being the high purity of the product, and the control of particle size is achieved by adjusting the current or the potential applied to the system. Cabrera *et al* prepared Fe_3_O_4_ NPs with sizes between 20 and 30 nm by Fe electro-oxidation in the presence of an amine surfactant, which acted as a supporting electrolyte and coating agent for particle size and aggregation control during the synthesis process. The distance between electrodes is critical for the successful synthesis of IONPs [112]. Recently, Rodríguez-López *et al* reported magnetic IONPs with controlled size distribution were electrochemically synthesized by applying a dissymmetric pattern of potential pulses to iron-based electrodes in aqueous media. It was found that Fe_3_O_4_ NP formation was favored, while avoiding the formation of metallic Fe particles with more anodic potentials and the longest time [113].

In fact, flow injection synthesis is a modified co-precipitation method. In the reaction process, different precursors can be added by pumping with a controllable flow rate. Therefore, this method showed some advantages, such as high reproducibility, high mixing homogeneity, and an opportunity for a precise external control of the process [108].

**Figure 10.**
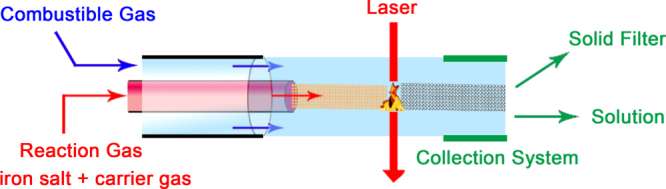
Detail of the reaction area where the laser interacts with the gas reactants and the influence of the collection system to obtain larger aggregates (solid filter) or well-dispersed ultrasmall IONPs (solution, the size is below 5 nm) under similar experimental conditions.

Spray and laser pyrolysis are the main aerosol technologies for fabricating magnetic IONPs. In spray pyrolysis, fine IONPs are produced by the evaporation of ferric salts, drying, and pyrolysis reaction of liquid drops (a reducing agent in organic solvent) inside a high temperature atmosphere, especially the flame-spray. Particle size and size distribution depend on the size and size distribution of liquid drops, and the evaporation process of a solvent and the property of the starting material. Recently, Abid *et al* reported IONPs with variable oxidation states by flame-spray pyrolysis, revealing that the different flame configurations are an important factor of the morphology and size control of the final IONPs [114]. For reducing the reaction volume, laser became the energy resource and heated a gaseous mixture of iron precursor and a flowing mixture of gas producing small, narrow size, and non-aggregated NPs in the pyrolysis process (as shown in figure 10). Importantly, laser pyrolysis can produce well-dispersed fine IONPs. For example, Costo *et al* have synthesized very high crystallinity, and ultrasmall NPs (<5 nm) with a rather spheroid morphology and exceptionally narrow particle size distributions through an optimized acid treatment. The dissolution of the disordered layer from the particle surface and further recrystallization of an iron polymer activated the surface and prepared the particles for further functionalization with bioactive ligands [115]. However, the final IONPs made by this process had a very broad size distribution due to the difficulty of obtaining a uniform size of initial droplets or gaseous mixture.

The characterization of the above mentioned synthetic methods are briefly summarized in table 1. In terms of simplicity of synthesis, classical co-precipitation is the preferred route. In terms of size and morphology control of IONPs, thermal decomposition seems the best method to develop IONPs smaller than 20 nm, and the hydrothermal or solvothermal method seems to be the most suitable for producing IONPs larger than 20 nm. As an alternative, the other methods can also be used to synthesize magnetic IONPs with a narrow size distribution and controllable morphology. However, the major difficulties in the synthesis of IONPs are still to control the size, shape, composition and size distribution on the nanoscale due to the fact that the aggregation and/or continuous growth of IONPs to minimize the overall surface free energy and magnetic interactions. The current methods often involve a number of different steps with multiple microstructural problems that may have a pernicious influence on the magnetic performance. Therefore, searching for a facile and flexible fabricated method to produce IONPs with the desired morphologies without aggregation is of extreme importance to realize the full potential of these materials in biomedical applications. Thus, looking for new routes of large-scale synthesis and improvement of the known ones should be continued.

**Table 1.** Summary comparison of the synthetic methods for producing magnetic IONPs.

Method	Reaction and conditions	Reaction temp. [°C]	Reaction period	Size distribution	Shape control	Yield
Co-precipitation	Very simple, ambient	20–150	Minutes	Relatively narrow	Not good	High/scalable
Thermal decomposition	Complicated, insert atmosphere	100–350	Hours-days	Very narrow	Very good	High/scalable
Hydro- or solvothermal synthesis	Simple, high pressure	150–220	Hours-days	Very narrow	Very good	High/scalable
Sol–gel and polyol method	Complicated, ambient	25–200	Hours	Narrow	Good	Medium
Microemulsion	Complicated, ambient	20–80	Hours	Narrow	Good	Low
Sonolysis or sonochemical method	Very simple, ambient	20–50	Minutes	Narrow	Bad	Medium
Microwave-assisted synthesis	Very simple, ambient	100–200	Minutes	Medium	Good	Medium
Biosynthesis	Complicated, ambient	Room temp.	Hours-days	Broad	Bad	Low
Electrochemical methods	Complicated, ambient	Room temp.	Hours-days	Medium	Medium	Medium
Aerosol/vapor methods	Complicated, insert atmosphere	>100	Minutes-hours	Relatively narrow	Medium	High/scalable

## 3. Surface functionalization of magnetic IONPs

An unavoidable problem associated with magnetic IONPs in the size range is their intrinsic instability over longer periods, which manifests in two main ways: (1) loss of dispersibility, where small NPs tend to aggregate and form large particles to reduce the surface energy; and (2) loss of magnetism, where bare IONPs are easily oxidized in air due to their high chemical activity, especially Fe_3_O_4_ and *γ*-Fe_2_O_3_ NPs. Therefore, it is crucial to develop a proper protection strategy to chemically stabilize bare IONPs against damage during or after the subsequent application. For biomedical applications, it is necessary to obtain water dispersible NPs, because most biological media are nearly neutral aqueous solutions.

**Figure 11.**
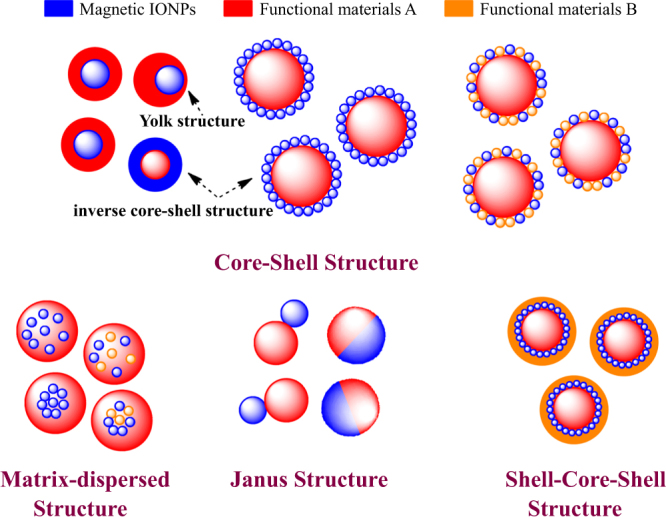
Typical morphologies of magnetic composite nanomaterials. Blue spheres represent magnetic IONPs, and the non-magnetic entities and matrix materials are displayed in other colors. The nonmagnetic entity may provide the composite material with further functionalities and properties, providing multifunctional hybrid systems.

In view of the many strategies and their subsequent application, efforts have been devoted to fabricating four types of IONP-based materials, including the core–shell structure, matrix dispersed structure, Janus-type heterostructures and shell–core–shell structure (figure 11).

### 3.1. Core–shell structure

In this structure, the iron oxide core was encapsulated in an inorganic or an organic coating that renders the whole particle stable and biocompatible, and may serve as a support for biomolecules. Generally, IONPs are not located at the center of the functional coating material; this structure is also known as a yolk structure. Indeed, the magnetic composite nanomaterials not only provide the material with an improved stability of the nanoparticulate building blocks, but also further introduce new physical and biological properties, and multifunctional behaviors. Thus, in the inverse core–shell structure, the magnetic IONPs will coat the surface of non-magnetic functional materials. Moreover, magnetic IONPs can combine one or more functional materials and further coat with another functional material on the functionalized surface. The above structures are collectively called core–shell structures.

However, some literature has reported ‘shell–core’ structures in magnetic nanomaterials; in this structure, the iron oxide will coat the surface of core materials [116–118]. For example, Zhan and Zhang have reported the synthesis of CdSe@Fe_2_O_3_ core–shell NPs by a one-step seeded-growth approach. These NPs not only retain their individual semiconducting and magnetic functionalities, but also exhibit some new properties that are affected by the coating components. These bi-functional CdSe@Fe_2_O_3_ NPs might find potential applications in biosensing and biomedical research [119].

### 3.2. Matrix-dispersed structure

Magnetic IONPs are dispersed in a matrix to prevent the superparamagnetic NPs from aggregating into large ferromagnetic species. Matrix-dispersed NPs can be created in a variety of different states, e.g. dispersed in a continuous amorphous matrix, grafted on larger, mesoscale particles, or well defined, three-dimensional superstructures of NPs [120].

### 3.3. Janus structure

In Janus structure, one side is magnetic IONPs, and the other side is functional materials. Anisotropic surface chemical compositions are interesting for applications even if one is not concerned with self-assembly. For example, Sun *et al* developed dumbbell-like Au-Fe_3_O_4_ and Pt- Fe_3_O_4_ NPs, where the sizes of the particles are tuned from 2 to 8 nm for Au and Pt, and 4 nm to 20 nm for Fe_3_O_4_ [121, 122]. This Janus particle can be used in target-specific platin delivery [123]. Zhao and Gao have prepared magnetic *γ*-Fe_2_O_3_ǁSiO_2_ Janus particles by flame synthesis. The highly uniform *γ*-Fe_2_O_3_ǁSiO_2_ presents excellent aqueous dispersibility, consequently providing different choices for further manipulation of Janus particles to form interesting assembled structures [124].

### 3.4. Shell–core–shell structure

In this structure, the location of magnetic IONPs is between the two functional materials. Most applications require magnetic IONPs to be embedded in the nonmagnetic layers to avoid aggregation and sedimentation of magnetic IONPs as well as to endow them with particular surface properties for specific applications. For example, luminescent layers, magnetic IONPs and biocompatible polymer layers are combined into bimodal nanocomposite materials, allowing manipulation by an external magnetic field and real time optical visualization at the same time.

A prerequisite for every possible applied structure is the proper surface protection or functionalization of such magnetic composite NPs, which determines their interaction with the environment. These interactions ultimately affect the colloidal stability of the composite particles, and may yield a controlled assembly or the delivery of NPs to a target, especially by appropriate functional organic materials or inorganic materials on the IONP surface [125].

### 3.5. Organic materials

IONPs with any organic material coating are used mainly for magnetic recording, electromagnetic shielding, MRI, and especially in the biological field for specific drug targeting, magnetic cell separation, etc. The stability of magnetic NPs under an external high applied magnetic field is very important for *in vivo* biological application as well as in other fields. Several approaches have been developed to functionalize IONPs, including *in situ* coatings and post-synthesis coating, which are the common routes for organic material coating on the IONP surface [126–128]. Furthermore, to stabilize the particles against aggregation and with good biocompatibility, the IONPs are coated with different organic materials, such as dextran, starch, poly(ethylene glycol) (PEG), poly (D, L-lactide) (PLA), polyethylenimine (PEI), especially for hydrophilic organic materials.

#### 3.5.1. Small molecules and surfactants

With proper surface modification, magnetic IONPs can be functionalized by special groups (e.g. –OH, –COOH, –NH_2_, –SH), which are suitable for further modifications by the attachment of different bioactive molecules for various applications.

As a small molecule, silane is often used to modify and endow the functionalized end groups to the surface of bare IONPs directly for post-connecting with metal ions, polymers, biomolecules or other biological entities. Significantly, silane modified magnetic IONPs still maintain the saturation magnetization values of the bare IONPs, where the decreased value is often less than 5 emu g^−1^; this character illustrates that the magnetic separation is not affected after silane modification. 3-aminopropyltriethyloxysilane (APTES), *p*-aminophenyltrimethoxysilane (APTS) and mercaptopropyltriethoxysilane (MPTES) agents are the most common silanes for anchoring the –NH_2_ and –SH, respectively. For instance, Shen *et al* reported a facile approach to synthesize APTES-coated magnetic IONPs (Fe_3_O_4_@APTES) with tunable surface functional groups for potential biomedical applications. The cytotoxicity and hemolytic assay results demonstrated that acetylation of the amine groups on the surfaces of IONPs would significantly improve the particles’ cytocompatibility and hemocompatibility [129]. Furthermore, as seen in our previous study, APTES was beneficial to maintaining the morphology of the Fe_3_O_4_ NPs, whereas MPTES modification caused a slight decrease in the saturation magnetization [130]. Additionally, the silane ligand-exchange reaction can make the hydrophobic IONPs change into water-dispersible NPs.

However, fabrication of oil-soluble type IONPs is very important for obtaining monodisperse IONPs. The most common organic compounds are oleic acid and oleyamine, which have a C18 tail with a cis-double-bond in the middle, forming a kink. Such kinks have been postulated as being necessary for effective stabilization, which can be a reasonable explanation for why stearic acid cannot stabilize IONPs (with no double-bond in its C18 tail) [1]. Moreover, oleic acid is widely used in IONP synthesis because it can form a dense protective monolayer, thereby producing highly uniform IONPs. Generally, the oleic acid and oleyamine are often used in the high-temperature thermal decomposition reaction process. For instance, Fe_3_O_4_ was synthesized via facile thermal decomposition of Fe(acac)_3_ in the presence of oleic acid or/and oleyamine. In a typical procedure, Fe(acac)_3_ is added to oleic acid and/or other organic compound (such as phenyl ether, 1, 2-hexadecane diol, etc) at room temperature. The reaction mixture was heated to >100 °C under a nitrogen atmosphere with vigorous stirring, and then kept at that temperature for a certain time. The above well-mixed solution was then heated to >300 °C, and the solution color gradually became black, indicating that the magnetic NPs were being formed in the presence of oleic acid and another organic compound [41, 131–133]. Salas *et al* have shown that the high temperature decomposition of an iron oleate complex can be used to obtain superparamagnetic nanocrystals with sizes over 10 nm, where the as-obtained IONPs exhibited high saturation magnetization. The results concerning the size of the IONPs as a function of the oleic acid added to the reaction medium showed a complex behavior that can be qualitatively explained in terms of the nucleation and growth rates. Broader size distributions lead to worse magnetic properties either in large (15 or 18 nm) or in small IONPs (9 nm) [134]. Moreover, oleic acid coating Fe_3_O_4_ NPs resulted in no appreciable changes in the overall magnetic behavior of the samples. These Fe_3_O_4_ NP systems with high values of *M*_S_, corresponding to 80% of the bulk value, are suitable for technological applications [135]. The final shape of the IONPs could be readily tuned from sphere to cube by adjusting the experimental parameters, such as reaction time, temperature, and surfactants [44].

Indeed, the magnetic IONPs resulting from high-temperature decomposition of an organic iron precursor are capped with nonpolar endgroups on their surface and are usually stable in nonpolar solvents (such as hexane). The capping molecules (also called ligands) are typically long-chain alkanes with polar groups binding to the IONPs’ surface. Hence, to take advantage of their high-quality properties in biological applications, it is necessary to transfer IONPs from organic phase to aqueous phase, and the hydrophobic surfactant coating needs to be replaced by a hydrophilic, biocompatible, and functional coating that allows controlled interaction with biological species.

To synthesize water-soluble magnetic IONPs directly, one way is to use small molecules (such as amino acid, citric acid, vitamin, cyclodextrin, etc) in the reaction process [136–138]. For example, Gao *et al* synthesized highly charged hydrophilic superparamagnetic Fe_3_O_4_ colloidal nanocrystal clusters with an average diameter of 195 nm by using a modified one-step solvothermal method. Anionic polyelectrolyte poly (4-styrenesulfonic acid-co-maleic acid) sodium salt (PSSMA) containing both sulfonate and carboxylate groups was used as the stabilizer. The PSSMA-stabilized IONP clusters could be well dispersed in water, phosphate buffered saline (PBS), and ethanol. Moreover, silica shells could be directly coated onto these clusters by the Stöber method. The colloidal nanocrystal clusters remained negatively charged in the experimental pH ranges from 2 to 11, and also showed high colloidal stability in PBS and ethanol [139]. Recently, Majeed *et al* reported a one-step protocol for the preparation of fairly monodisperse and highly water-soluble magnetic IONPs through a co-precipitation method using a novel multifunctional, biocompatible and water-soluble polymer ligand dodecanethiol-polymethacrylic acid (DDT-PMAA). The as-prepared IONPs were conjugated with the anti-cancer drug doxorubicin (DOX) and its efficacy, as a model drug delivery system, was determined using HepG2 cells. The efficiency of the drug-NP conjugates i.e., covalently bound DOX-IONPs and electrostatically loaded DOX/IONPs, was found to be significantly higher than that of the free drug (DOX). Indeed, owing to the several intrinsic properties of DDT-PMAA, it not only efficiently controls the size of the IONPs but also gives them excellent water solubility, long time stability against aggregation and oxidation, biocompatibility, and a multifunctional surface rich in thioether and carboxylic acid groups [140]. Obviously, these highly colloidal stable IONPs have potential applications in biotechnology.

**Figure 12.**
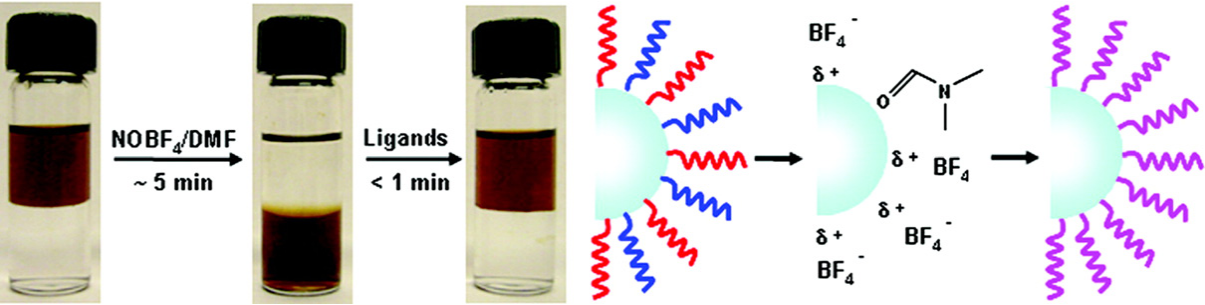
A facile ligand-exchange approach, which enables sequential surface functionalization and phase transfer of colloidal NCs while preserving the NC size and shape. (Reprinted with permission from A Dong *et al* 2010 *J. Am. Chem. Soc.* **133** 998. Copyright 2010 American Chemical Society.)

Another way is to use a ligand exchange procedure to change the polarity of the hydrophobic layer to being hydrophilic [141–143]. It involves adding an excess of ligand to the nanoparticle solution, resulting in the displacement of the original ligand on the surface of NPs. For instance, Dong *et al* reported a facile ligand-exchange approach, which is enabled for sequential surface functionalization and phase transfer of colloidal IONPs while preserving the NPs’ size and shape. Nitrosonium tetrafluoroborate (NOBF_4_) is used to replace the original organic ligands attached to the NPs’ surface, stabilizing the NPs in various polar and hydrophilic media for years, without aggregation or precipitation (shown in figure 12). Significantly, as illustrated in figure 12, the hydrophilic NPs obtained by NOBF_4_ treatment can readily undergo secondary surface modification due to the weak binding affinity of BF_4_^−^ anions to the surface of NPs, allowing fully reversible phase transfer of NPs between hydrophobic and hydrophilic media [144]. Ninjbadgar and Brougham have reported a novel and efficient method to produce water dispersible superparamagnetic Fe_3_O_4_ NPs by ring opening coupling reactions. Fe_3_O_4_ NPs prepared by non-hydrolytic organic phase methods were subsequently functionalized with (3-glycidyloxypropyl) trimethoxysilane, the linker between the Fe_3_O_4_ NPs and organic molecule prevent aggregation, and it also is available for subsequent coupling reactions with a wide range of polymers and biomolecules. Ring opening coupling reactions were used to coat the epoxy-functionalized Fe_3_O_4_ NPs with aminated polymers (polyetheramines) or small molecules (arginine). The obtained NPs, with hydrodynamic size of 13 nm, are found to be very stable over extended periods in water or PBS due to the presence of a dense stabilizer layer covalently anchored onto the surface. Exceptionally high spin-lattice relaxivity, low *r*_2_/*r*_1_ ratios were exhibited in the clinical MRI frequency range, irrespective of the molecule selected for nanoparticle stabilization. As a result, the dispersions are excellent candidates for incorporation into multifunctional assemblies or for use as a positive contrast agent for MRI [145].

#### 3.5.2. Polymers

Compared with small molecules and surfactants, polymer functionalization not only provides multifunctional groups and more colloid stability, but also plays a significant role regarding its biological fate (i.e., pharmacokinetics and biodistribution) [146]. Furthermore, a large number of natural and synthetic biodegradable polymers, such as polyaspartate [147], polysaccharides [148–150], gelatin [151–153], starch [154–156], alginate [157–159], poly(acrylic acid) [160–162], PEG [163, 164], poly(D,L-lactide) (PLA) [165–167], chitosan [168, 169], and polymethylmethacrylate (PMMA) [170–172], are currently under evaluation for the functionalized materials of IONPs.

Several approaches have been developed to functionalize IONPs with polymers, where the common approaches include *in situ* and post-synthesis coating. In the *in situ* approach, the conventional routes are mini/micro-emulsion polymerization and the sol–gel process for polymer functionalizing IONPs (polymer@IONPs) during the synthesis process [173, 174]. The organic molecules capped the IONPs and formed a capping layer during the emulsion polymerization process; the conventional structure is a core–shell structure or matrix dispersed structure [75, 175]. Unfortunately, these direct surface modification strategies are often unsuccessful in maintaining colloidal stability and the thickness of the shell is not easy to control.

Consequently, the prevalent routes for polymer@IONPs is post-synthesis functionalization, which is based on the pre-prepared IONPs for further polymer functionalization via a one-pot route, self-assembly, or heterogeneous polymerization (such as inverse mini/emulsion polymerization and dispersion polymerization) [171]. Particularly, the one-pot method is a facile route for obtaining polymer@IONP composite nanomaterials [176]. The physical adsorption and functional groups anchoring on the surface of IONPs are the common mechanism in this strategy, the resulting structure of complex NPs is prone to form a core–shell structure. Furthermore, the covalent bonding is a wide and commonly used functional technique and the cross-linking is made by using the alkyl chain or carboxylic acid functionalized thiol and hydrogen bonding [177]. In addition, various heterogeneous polymerizations with water-soluble monomers have been explored to prepare well-defined core–shell or matrix dispersed structure polymer@IONPs for biomedicine applications [178, 179]. For instance, an all-in-one NP platform with a size-range of 30 nm–100 nm was developed based on an oil-in-water emulsion method. The hydrophobic layer coated IONPs were included in the soybean oil core of the emulsions. Subsequently, these oil droplets are stabilized by a PEGylated lipid mixture to favor the formation of small particles, which increased the longevity of the complex particles in circulation, so the complex NPs are enabled for MRI detection. The emulsions allowed loading high quantities of iron oxide nanocrystals, and the resulting complex particles caused a remarkably high transverse relaxivity (*r*_2_) [180].

**Figure 13.**
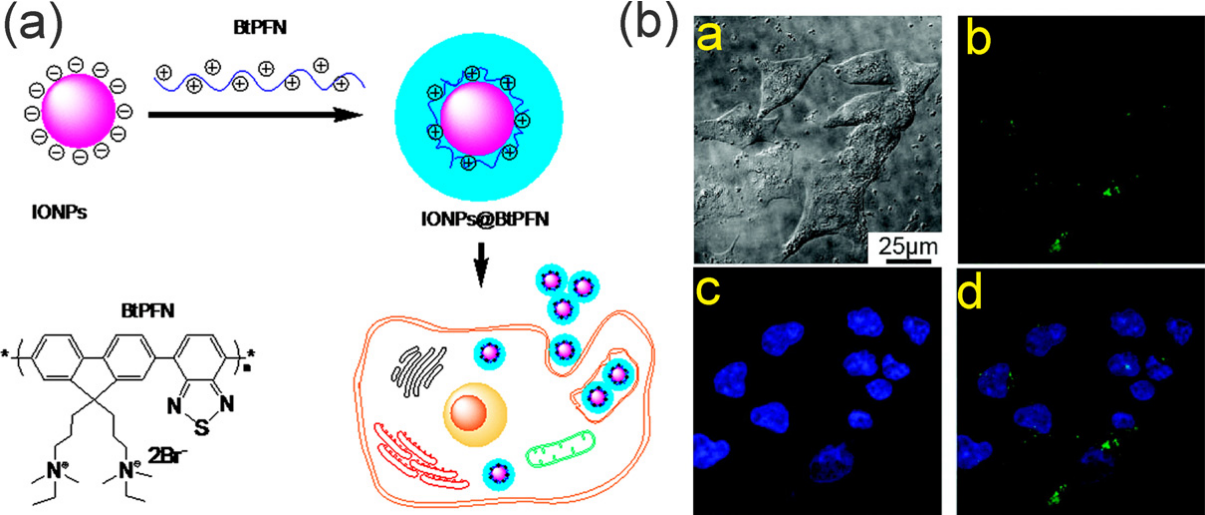
Schematic of the preparation of IONP@conjugated polymer (BtPFN) and internalization by cancer cells; confocal laser scanning microscopy (CLSM) images of Bel-7402 cells incubated with MP/BtPFN (green color) for 4 h at 37 °C, whereas cell nuclei are stained by Hoechst 33342 dye (blue color). (a) Bright-field image. (b)–(d) Fluorescence images of the green (b) and blue (c) channels, and a merged image (d). (Reprinted with permission from B Sun *et al* 2010 *Macromolecules* **43** 10348. Copyright 2010 American Chemical Society.)

The stability of IONPs can be enhanced and the application field extended by introducing polymers with multiple functional groups. For example, conjugated polymers, which are characterized by a delocalized electronic structure, exhibited efficient coupling between optoelectronic segments, thereby the conjugated polymer functionalized IONPs can be applied in imaging, diagnosis, and therapy [181, 182]. Wang *et al* used the fluorescent conjugated polyelectrolyte (BtPFN) to coat the surface of magnetic IONPs and form IONP/BtPFN composite NPs with a positively charged fluorescent shell by electrostatic adsorption (as shown in figure 13). The organic/inorganic hybrid NPs display a simultaneous response toward light excitation and external magnetic fields. Furthermore, these nanocomposites can be used as robust fluorescent probes in cell imaging, and if optimized, as multicolor probes to detect interactions of tremendous NPs with living cells. The long-term effects of IONP/BtPFN NPs in cell indicated most MP/BtPFN NPs were clearly in the cytoplasm, whereas a few of them migrated to the region very close to the outer nuclear membranes of the cells [183].

Presently, the fashionable trend for polymer functionalized IONPs in biomedicine is functionalizing with smart polymers, which endow special properties to IONPs for a stimulus response environment, such as pH, temperature, light, etc [184, 185]. Generally, pH-sensitive polymers are polyelectrolytes that bear in their structure weak acidic or basic groups that either accept or release protons in response to changes in environmental pH. Thermo-sensitive polymers can be classified into different groups depending on the mechanism and chemistry of the groups. The functional groups of polymers have a crucial role in stimulus response properties and comprehensive application in biomedicine such as drug delivery, MRI, and biosensors; some typical polymers and functional groups are listed in table 2 [186–188].

**Table 2.** Examples of smart polymer functionalized IONPs.

Type of stimulus	Polymers	Functional groups	Clinical products and examples	Refs.
pH	Polypropylacrylic acid (PPAA), polyethacrylic acid (PEAA); Poly(methyl methacrylate) (PMMA) Poly(acrylic acid) (PAA)	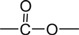		[192, 193]
	Chitosan	–NH_2_ –OH		[194]
	Poly(L-lysine), Poly(ethyleneimine) (PEI),	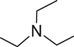 –NH– –NH_2_		[195, 196]
	Poly(4-vinylpyridine), poly(2-vinylpyridine) (PVP) and poly(vinylamine) (PVAm)	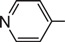 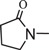		[197, 198]
Temperature	Poly(N-isopropylacrylamide) PNIPAAm	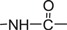	Pluronics® F127 Poloxamers® 407, Tetronics®	[199, 200]
	Poly(N,N′-diethyl acrylamide), Poly(dimethylamino ethyl methacrylate)	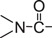 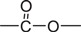 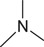	PEG/PLGA, Regel ®	[201]
	Polyethylene glycol (PEG)	–O–, –OH	T_1_ MR Contrast Agent	[202]
Light	Polyethylene glycol (PEG)	–O–		[203]
	Poly (lactic acid)	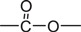		[204]

Additionally, considerable interest has been attracted to functionalizing IONPs with amphiphilic block copolymers, which incorporate more functional groups into the polymers for multifunctional applications [189]. The self-assembly method is the common route to design and prepare stable complex IONPs with amphiphilic block copolymers in the liquid phase. Furthermore, this technique is used to prepare film platforms, drug carriers and MRI [190]. For instance, novel multifunctional nanocomposites were successfully prepared through a simple self-assembly process for the controlled release of anticancer drug and MRI. The SPIONPs were ‘fixed’ between the hydrophobic segment of the pH-sensitive amphiphilic polymer (HAMAFA-b-DBAM) and the surface of hollow mesoporous silica NPs (HMS), which were modified by the long-chain hydrocarbon octadecyltrimethoxysilane. The amphiphilic polymer was further conjugated with a folic acid (FA) group; the nanocomposites could target the FA receptor of over-expressed tumor cells efficiently. The loaded drug can be released from the HMS core triggered by the mildly acidic pH environment in the cancer cells due to the hydrolysis of the pH-sensitive polymer shell. The targeting process of the nanocomposites could be easily tracked by MRI due to the magnetism of the SPIONPs [191].

However, it is worth noting that, in some cases, the presence of polymer or copolymer layers may negatively influence the magnetic properties of the IONPs. Thus, great caution has to be exercised during the selection of polymeric materials for the stabilization of magnetic colloids.

#### 3.5.3. Biomolecules

Recently, biomolecule functionalized magnetic IONPs have become a common and effective strategy in the biological separation, detection, sensor and other bio-applications due to their higher biocompatibility. The various biomolecules, including enzymes, antibodies, proteins, biotin, bovine/human serum albumin, avidin and polypeptides have been bound onto the surface of IONPs [7, 205–209].

**Figure 14.**
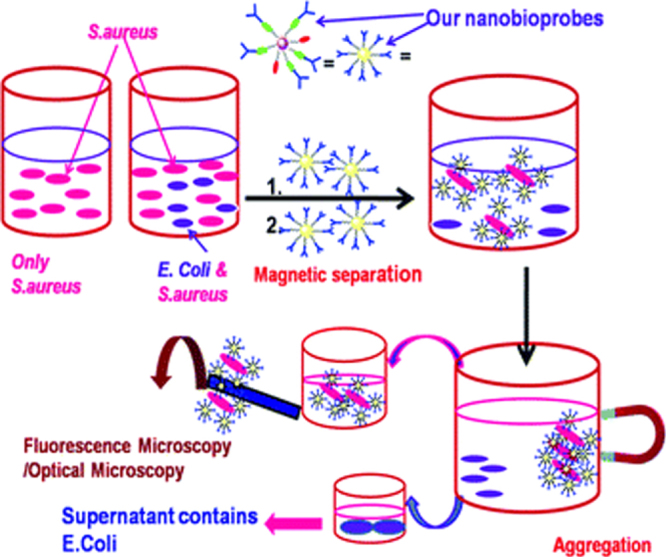
Schematic demonstration of pathogen detection by antibody-modified-fluorescent-MPA–Au–Fe_3_O_4_ nanocomposites. (Reprinted with permission from D Bhattacharya *et al* 2011 *J. Mater. Chem.* **21** 17273. Copyright 2011 Royal Society of Chemistry.)

For instance, Magro *et al* reported on the surface characterization, functionalization, and application of stable water suspensions of novel surface active maghemite NPs by avidin. Bound avidin was determined by measuring the disappearance of free avidin absorbance at 280 nm, as a function of increasing nanoparticle concentration, showing the presence of 10 ± 3 avidin molecules per nanoparticle. Fe_2_O_3_@avidin was applied for the large scale purification of recombinant biotinylated human sarco/endoplasmic reticulum Ca^2+^-ATPase (hSERCA-2a), expressed by *Saccharomyces cerevisiae*. The protein was magnetically purified, and about 500 *μ*g of a 70% pure hSERCA-2a were recovered from 4 L of yeast culture, with a purification yield of 64% [210]. As shown in figure 14, Bhattacharya *et al* demonstrated a rapid, sensitive, specific and efficient method for the detection of *Staphylococcus aureus* (*S*. *aureus*) as the model analyte at ultra-low concentrations using antibody labeled multifunctional Au-Fe_3_O_4_ nanocomposites. Fluorescence/confocal as well as optical microscopy could detect a total count of *S*. *aureus* within concentrations of 10^2^–10^7^ CFU mL^−1^ in 30 min and the detection limit is 10^2^ CFU mL^−1^. These antibody targeted NPs are a potent probe for a broad application in detecting specific bacteria, *S*. *aureus*, in various biodetection systems [211]. The biological molecule functionalized IONPs will greatly improve the particles’ biocompatibility. Such magnetic IONPs can be very useful to assist an effective separation of proteins, DNA, cells, biochemical products, etc.

### 3.6. Inorganic materials

Inorganic materials can possess a number of different properties such as high electron density and strong optical absorption (e.g. noble metal particles, in particular Au and Ag), photoluminescence in the form of fluorescence (semiconductor quantum dots, e.g. CdSe or CdTe) or phosphorescence (doped oxide materials, e.g. Y_2_O_3_), or magnetic moment (e.g. manganese or cobalt oxide NPs) [212–215]. These coatings not only provide stability to the NPs in solution but are also widely used for the improvement of semiconductor efficiency, information storage, optoelectronics, catalysis, quantum dots, optical bioimaging, biological labeling, and so on. Especially, some inorganic materials help in binding various biological ligands to the IONP surface, such as silica, Au, metal oxides, etc [216–218].

#### 3.6.1. Silica

Silica-coated IONPs (IONP@silica) is a classical and important composite material for both fundamental study and bio-applications. Silica coating can enhance the dispersion in solution because the silica layer could screen the magnetic dipolar attraction between magnetic IONPs. Additionally, the silica coating would increase the stability of IONPs and protect them in an acidic environment. Finally, owing to the existence of abundant silanol groups on the silica layer, IONP@silica could be easily activated to provide the surface of NPs with various functional groups. For practical applications, it is required that each IONP should be coated with a homogeneous silica layer without core-free silica particles, regardless of the size of the NPs. For instance, as a heating source and magnetic guidance, IONPs play an important role in hyperthermia and targeted drug delivery, and the existence of core-free silica particles will lead to a loss in the effective dose of IONPs. The major reason causing uneven heating in hyperthermia and tissue distribution of the targeted drugs can be attributed to the unequal core number and silica shell thickness.

Three different approaches have been explored to generate IONP@silica nanomaterials. The first method relied on the well-known Stöber process [219], in which silica was formed *in situ* through the hydrolysis and condensation of a sol–gel precursor, this is a prevailing choice for preparing IONP@SiO_2_. Generally, the IONPs were homogeneously dispersed in the alcohol, then the silane was added, and finally the water or ammonia aqueous solution was dropped into the mixed solution and IONP@SiO_2_ formed. Tetraethoxysilane (TEOS), vinyltriethoxysilane (VTEOS), octadecyltrimethoxy silane are the most common used silanes, which easily bind on the surface of IONPs through OH groups [220–222]. For example, Xuan *et al* synthesized monodispersed *γ*-Fe_2_O_3_@meso-SiO_2_ by this method, and the size of the magnetic core and the thickness of the porous shell were controlled by tuning the experimental parameters. The magnetic property of the *γ*-Fe_2_O_3_ porous core enabled the microspheres to be used as a contrast agent in magnetic resonance imaging with a high *r*_2_ (76.5 s^−1^ mM^−1^ Fe) relaxivity. The biocompatible composites possess a large BET surface area (222.3 m^2^ g^−1^); the composite NPs have been used as a bi-functional agent for both MRI and drug carriers [223]. In our previous report, the ultrafine hollow Fe_3_O_4_/silica NPs (the diameter of about 32 nm) with a high surface area were synthesized by using CTAB and AOT as co-templates and subsequent annealing treatment. The composite NPs can be magnetic separated by the external magnetic field [224]. The iron oxide@SiO_2_ NPs are often used in MRI; it is noteworthy that the increase of the silica coating thickness will cause a significant decrease of the *r*_1_ and *r*_2_ relaxivities of their aqueous suspensions [225]. In this method, the amount of silane used is a key factor for tuning the silica shell thickness, and the adequate silica shell thickness can therefore be tuned to allow for both a sufficiently high response as a contrast agent, and adequate grafting of targeted biomolecules [226].

**Figure 15.**
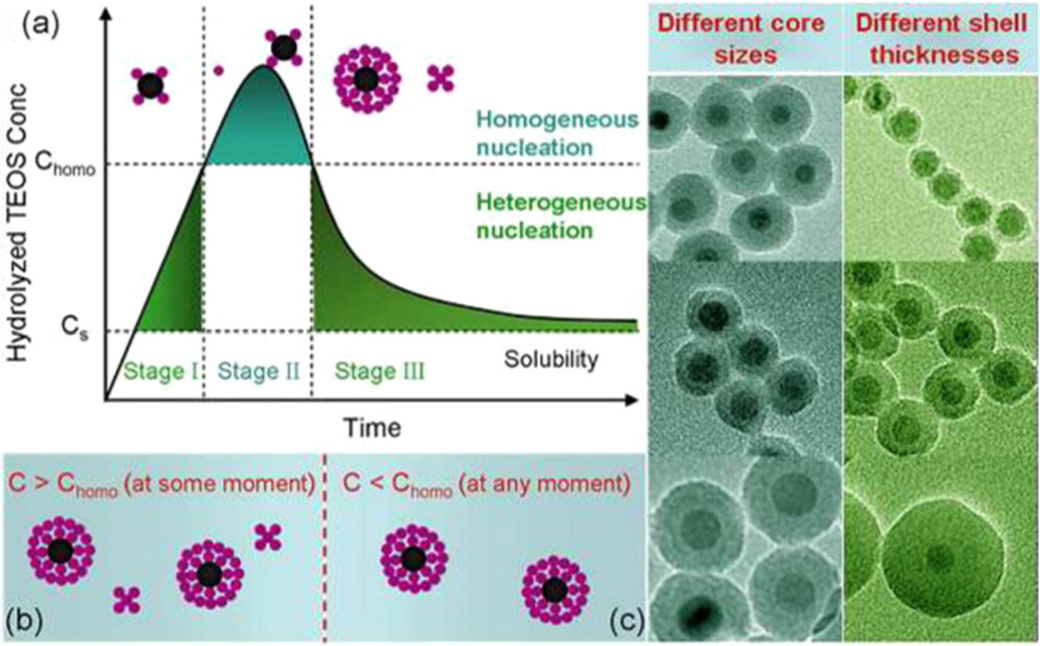
(a) La Mer-like diagram: hydrolyzed TEOS (monomers) concentration against time on homogeneous nucleation and heterogeneous nucleation, (b) the existence of Fe_3_O_4_@SiO_2_ core/shell NPs and SiO_2_ NPs in the reaction production when C > C_homo_ at some moment, (c) only the existence of Fe_3_O_4_@SiO_2_ core/shell NPs in the reaction production when C < C_homo_ at any moment. (Reprinted with permission from H L Ding *et al* 2012 *Chem. Mater.* **24** 4572. Copyright 2012 American Chemical Society.)

The second method was based on microemulsion synthesis, in which micelles or inverse micelles were used to confine and control the coating of silica on core NPs [227]. It is noteworthy that this method requires much effort to separate the core–shell NPs from the large amount of surfactants associated with the microemulsion system. Recently, Ding *et al* reported the coating regulations of Fe_3_O_4_ NPs by the reverse microemulsion method to obtain Fe_3_O_4_@SiO_2_ core–shell NPs. As shown in figure 15, the regulation produces core–shell NPs with a single core and with different shell thickness and especially it can be applied to different sizes of Fe_3_O_4_ NPs and avoid the formation of core-free silica particles. The small aqueous domain was suitable to coat ultrathin silica shell, while the large aqueous domain was indispensable for coating thicker shells. To avoid the formation of core-free silica particles, the thicker silica shells were achieved by increasing the content of either TEOS through the equivalently fractionated drops or ammonia with a decreased one-off TEOS [228]. The advantage of this method is that uniform silica shells with controlled thickness on the nanometer scale can be realized.

The third method is aerosol pyrolysis, in which IONP@SiO_2_ were prepared by aerosol pyrolysis of a precursor mixture composed of silicon alkoxides and metal compound in a flame environment [229]. For instance, Basak *et al* have reported the controlled synthesis of a core–shell type *γ*-Fe_2_O_3_/SiO_2_ nanocomposite, which is demonstrated in a single step in a furnace aerosol reactor using premixed precursors. The result reveals that the synthesis of a silica-coated *γ*-Fe_2_O_3_ nanocomposite depends on the choice of proper precursors as proposed in the generalized mechanism [230].

#### 3.6.2. Carbon

Carbon protected IONPs have recently triggered enormous research activities due to their good chemical and thermal stability, and intrinsic high electrical conductivity. The carbon coating provides an effective oxidation barrier and prevents corrosion in magnetic core materials. Hydrophilic carbon coating on iron oxide nanoparticle cores endows better dispersibility and stability than those shown by bare IONPs [231].

**Figure 16.**
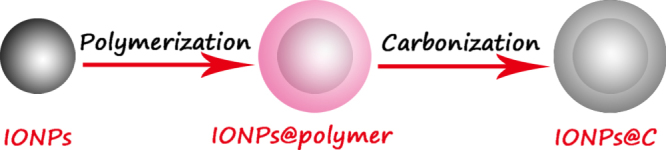
Schematic illustration of the fabrication of IONP@C composites.

Various approaches have been developed for synthesizing IONP@C core–shell nanostructures. As shown in figure 16, the common approach is a three-step process: firstly, magnetic IONPs are prepared as seeds by various methods, and then the polymer is coated through the polymerization process, finally forming IONP@C composite materials by annealing treatment. For example, Lei *et al* demonstrated that through a controlled coating of a thin layer of polydopamine on the surface of *α*-Fe_2_O_3_ in the dopamine aqueous solution, followed by subsequent carbonization, N-doped carbon-encapsulated magnetite has been synthesized and displayed excellent electrochemical performance as an anode material for lithium-ion batteries [232]. Li *et al* reported for the first time for the selected-control, large-scale synthesis of monodispersed Fe_3_O_4_@C core–shell spheres, chains, and rings with tunable magnetic properties based on structural evolution from eccentric Fe_2_O_3_@poly(acrylic acid) core–shell NPs [233].

Recently, much attention has been paid to the synthesis of Fe_3_O_4_/graphene as a new kind of hybrid material, owing to its wide-ranging applications in lithium-ion batteries, ion removal, sensors, catalysts, etc [234–237]. The unique properties of Fe_3_O_4_/graphene hybrids, combining effects from graphene, which has high conductivity and a large surface-to volume ratio, and Fe_3_O_4_ NPs, with their high magnetism, low price, and environmentally benign nature, have opened a new window for fabricating highly stable multifunctional nanomaterials [238]. Many approaches can be used to synthesize the Fe_3_O_4_/graphene hybrid materials. For example, Liu *et al* reported a superparamagnetic reduced graphene oxide-Fe_3_O_4_ hybrid composite (rGO-Fe_3_O_4_), which was prepared by the solvothermal reaction of Fe(acac)_3_ and graphene oxide (GO) in ethylenediamine (EDA) and water [239]. Zhang *et al* have described a facile approach to control the assembly of monodisperse Fe_3_O_4_ NPs on chemically rGO. First, reduction and functionalization of GO by PEI were achieved simultaneously by simply heating the PEI and GO mixture at 60 °C for 12 h. Meso-2,3-dimercaptosuccinnic acid (DMSA)-modified Fe_3_O_4_ NPs were then conjugated to the PEI moiety which was located on the periphery of the GO sheets via the formation of amide bonds between COOH groups of DMSA molecules bound on the surface of the Fe_3_O_4_ NPs and amine groups of PEI [240].

**Figure 17.**
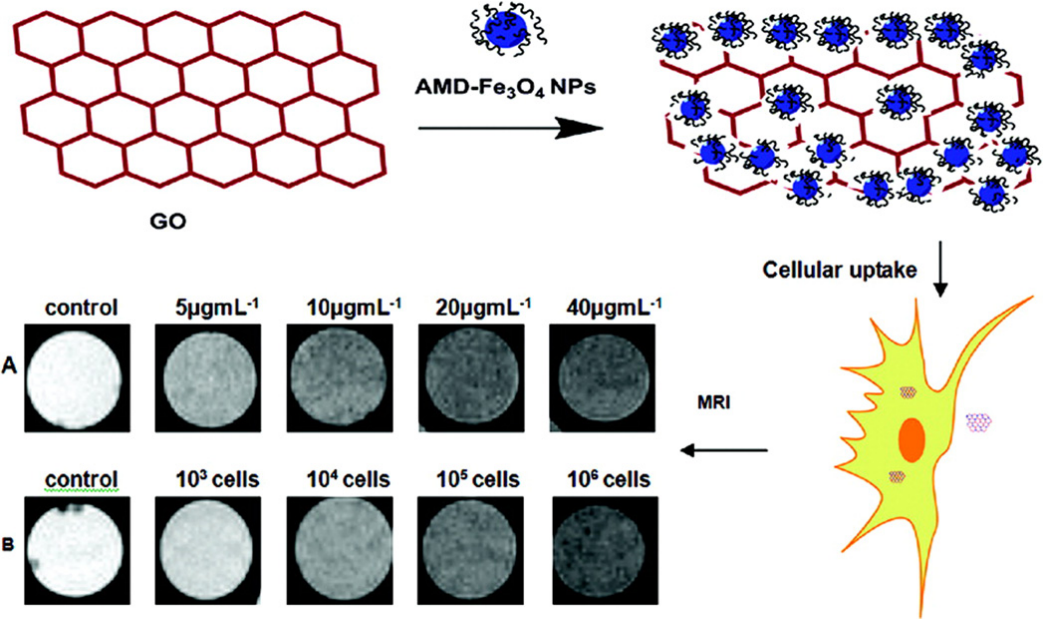
Schematic diagram showing preparation of Fe_3_O_4_–GO composites and using for cellular MRI. (Reprinted with permission from W H Chen *et al* 2011 *ACS Appl. Mater. Interfaces* **3** 4085. Copyright 2011 American Chemical Society.)

Moreover, Fe_3_O_4_/graphene hybrid materials have been used in biological fields, such as targeted drug delivery and MRI [241–243]. For example, Chen *et al* have reported that the fabricated composites of aminodextran-coated Fe_3_O_4_ NPs and GO were efficient for cellular MRI. As shown in figure 17, the *in vivo* study showed that the internalization of Fe_3_O_4_–GO composites has no effect on the cellular viability and proliferation. Compared to the bare Fe_3_O_4_ NPs, the Fe_3_O_4_–GO composites exhibit a significantly improved *T*_2_ weighted MRI contrast, which is explained by the fact that the Fe_3_O_4_ NPs formed aggregates on the GO sheets, resulting in a considerable enhanced *T*_2_ relaxivity [244].

#### 3.6.3. Metal

Metallic NPs (e.g., Au, Ag, Cu, Pd, Co, Pt, etc) possess a range of fascinating properties (localized surface plasmon resonance (LSPR) and surface-enhanced Raman scatting (SERS)) [245] and many anisotropic metallic NPs have been applied in catalysis [246], contrast imaging [247], medicine [248], and sensing [249]. The combination of metallic NPs and magnetic IONPs has also attracted increasing interest to materials scientists due to their combined physicochemical properties and potential properties in catalysts [250], biotechnology [251], and biomedicine [252, 253]. Generally, monodispersed iron oxide/metal nanostructures, such as core–shell, core–satellites, and dumbbell structures, exhibit binary/polynary properties. Moreover, these structures can be modified with different charges, functional groups or moieties on the surface of IONPs to improve stability and compatibility [254–256]. One of the most efficient and facile functionalization methods is the sequential growth of metallic components (e.g., Ag or Au) onto the surface of the IONP core in a one-pot reaction. The core–shell, core–satellites and dumbbell structures can be formed by microemulsion and thermal decomposition methods [257, 258]. However, it was found that the direct coating of IONPs with metal (or coating metal with IONPs) is very difficult in thermal decomposition, due to the dissimilar nature of the two surfaces and lattice [259]. Gold and silver seem to be ideal coatings owing to their low reactivity with IONPs. Furthermore, the introduction of surfactants and additives to the synthesis can alter the stability and surface property of IONPs or metallic NPs. Oleylamine (OA) is the common agent acting as capping agent, stabilizer and reductant [260]. For instance, OA and oleic acid capped 10 nm Fe_3_O_4_ NPs were synthesized via thermal decomposition of iron (III) oleate and were used for the synthesis of Fe_3_O_4_–Au NPs. Subsequently, core–shell Fe_3_O_4_–Au and Fe_3_O_4_–Au–Ag NPs are prepared by depositing Au and Ag on the surface of Fe_3_O_4_ NPs. Furthermore, the tunable plasmonic property of the core–shell structure was adjusted by shell thickness to be either red-shifted (to 560 nm) or blue-shifted (to 501 nm), which has great potential for nanoparticle-based diagnostic and therapeutic applications [261].

Another common route to synthesize IONP/metal composites involves multi-step methods including the seed-mediated method and emulsion method, resulting in core–shell, aggregated, and multilayers or hybrid IONP/metal structures. The most commonly reported structure in the literature is the core–shell Fe_3_O_4_@Au structure. For instance, monolayer-capped core–shell Fe_3_O_4_@Au was prepared by using the prepared Fe_3_O_4_ NPs as the seed and subsequently reducing Au ions. The Fe_3_O_4_ NP seeds displayed high efficiency of gold coating on the Fe_3_O_4_ NP seeds for formatted Fe_3_O_4_@Au core–shell NPs with controllable surface properties [262]. Layer-by-layer self-assembly is another feasible multi-step method for preparing multilayers or hybrid IONP/metal structures [258, 263]. The self-assembly approach renders NPs or other discrete components to spontaneously organize into ordered structures by molecular interactions [264], and the chemistry conjugation, organic surfactants with aliphatic/hydrophobic tail groups, and/or hydrocarbon solvents are the primary interactions for the formation of hybrid IONP/metal structures [258, 265]. For example, FeOOH–Au hybrid nanorods were synthesized by a layer-by-layer technique and subsequently those hybrid nanorods can be transformed into Fe_2_O_3_–Au and Fe_3_O_4_–Au hybrid nanorods via the controllable annealing process. The homogenous deposition of Au NPs onto the surface of FeOOH nanorods is attributed to the strong electrostatic attraction between metal ions and polyelectrolyte-modified FeOOH nanorods [266]. Recently, Truby *et al* prepared hybrid plasmonic–superparamagnetic NPs (gold nanorods–superparamagnetic IONPs, Au NR–SPIONs) with unique optical and magnetic properties by a facile aqueous-based, self-assembly approach. In this process, although only Au NRs were functionalized by carboxyl-bearing surface ligands, the hybrid Au NR–SPION nanostructures were produced upon simple mixing of the components owing to the chemisorption between the carboxyl groups and SPION surface. This hybrid SPION–Au NP structure maintained similar plasmonic properties to COOH–Au NPs according to the extinction spectra [190].

**Figure 18.**
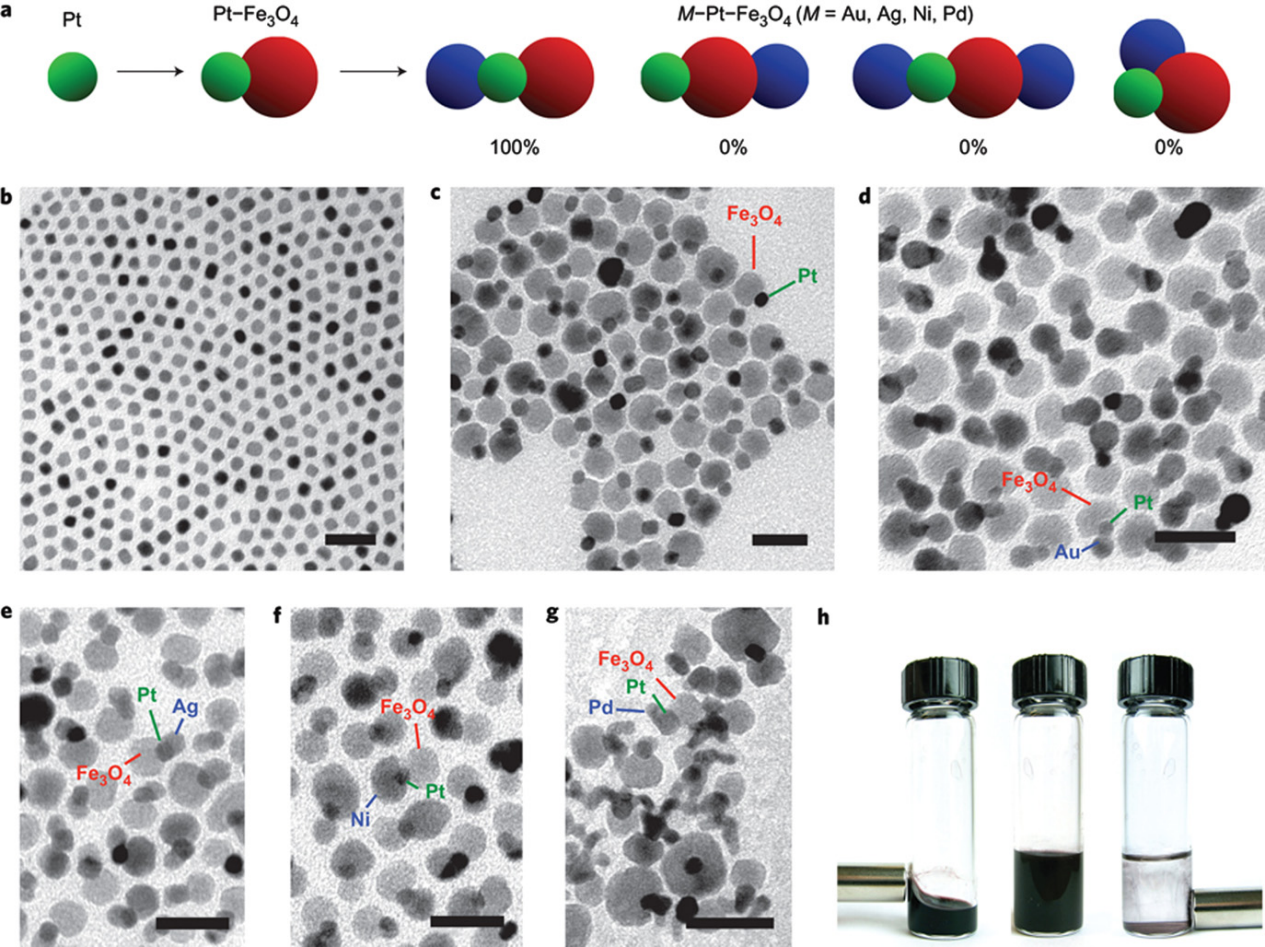
Stepwise construction of M–Pt–Fe_3_O_4_ heterotrimers (M = Ag, Au, Ni, Pd). a, Schematic showing the multistep synthesis of M–Pt–Fe_3_O_4_ heterotrimers, along with the most significant possible products and their observed frequencies (expressed as the percentage of observed heterotrimers, not total yield). Representative TEM images show Pt nanoparticle seeds (b), Pt–Fe_3_O_4_ heterodimers (c) and Au–Pt–Fe_3_O_4_ (d), Ag–Pt–Fe_3_O_4_ (e), Ni–Pt–Fe_3_O_4_ (f) and Pd–Pt–Fe_3_O_4_ (g) heterotrimers. All scale bars are 25 nm. (h) Photographs of a vial that contains Au–Pt–Fe_3_O_4_ heterotrimers in hexane (left), which responds to an external Nd–Fe–B magnet, the same vial with Au–Pt–Fe_3_O_4_ heterotrimers in a larger volume of hexanes (middle) and the same vial after precipitation of the heterotrimers with ethanol (right). The precipitated heterotrimers collect next to the external magnet. (Reprinted with permission from M R Buck *et al* 2011 *Nat. Chem.* **4** 37. Copyright 2011 Nature Publishing Group.)

In all the above mechanisms, the formation of IONP/metal structures was via molecular or charged links between IONPs and metal, whereas the dumbbell IONP/metal structures interfacial interaction originate from electron transfer across the nanometer contact at the interface of IONPs and metal NPs, inducing new properties that are not present in the individual component [267]. Sun *et al* developed a series of dumbbell IONP/metal structures through controlling the nucleation and growth of only one Fe_3_O_4_ on each Au (or Pt, or Pd) seeding NPs under the current synthetic conditions, which was attributed to the possible electron transfer between Au and Fe, and applied for enhanced catalysis, target-specific imaging and delivery [121, 123, 268, 269]. Furthermore, they based this on the understanding of the formation mechanism and took insight from the mechanical property of dumbbell-like Au–Fe_3_O_4_ NPs by overgrowing Au_2_ on Au_1_–Fe_3_O_4_ NPs. The ‘tug-of-war’ mechanism between Au_2_ and Fe_3_O_4_ was attributed to the formation of a Au_2_–Au_1_–Fe_3_O_4_ ternary nanostructure after Au_2_ growing on the preformed Au_1_–Fe_3_O_4_ NPs. The strain energy between Au_1_ and Fe_3_O_4_ played an important role, which not only decided their structure stability, but might also affect their functional performance. The reduced compressive stress in Au_1_ NP will result in possibly unbalanced stress across the interface. As a result, Au_2_ extracted Au_1_ out from the Au_1_–Fe_3_O_4_ conjugation, generating a new dumbbell-like Au_2_–Au_1_ and a dented Fe_3_O_4_ NP [270, 271]. As shown in figure 18, Buck *et al* have developed a total-synthesis framework for the construction of hybrid nanoparticle architectures that include M–Pt–Fe_3_O_4_ (M = Au, Ag, Ni, Pd) heterotrimers, M_x_S–Au–Pt–Fe_3_O_4_ (M = Pb, Cu) heterotetramers and higher-order oligomers based on the heterotrimeric Au–Pt–Fe_3_O_4_ building block. This synthetic framework conceptually mimics the total-synthesis approach used by chemists to construct complex organic molecules. The reaction toolkit applies solid-state nanoparticle analogues of chemoselective reactions, regiospecificity, coupling reactions and molecular substituent effects to the construction of exceptionally complex hybrid nanoparticle oligomers [272].

However, taking into account what the application requires and the relatively limited chemical stability of the metal-coated IONP core–shell, aggregated, and dumbbell, most studies have focused on the development of multi-layer IONP/metal components. Affinity via amine/thiol terminal groups, surfactants and biocompatibility multi-layers are applied to further enhance protecting the core from oxidation and corrosion, and to exhibit good biocompatibility [273–275]. Generally, co-polymers and branched polymers with biocompatibility are selected as effective stabilizers for metal–iron oxide composites. For instance, Lim *et al* utilized three different macromolecules, Pluronic F127, cationic polyelectrolyte poly(diallyldimethylamonium chloride) (PDDA), and PEG, to coat the preformed core–shell iron oxide/Au particles and promote colloidal stability in elevated ionic strength media. The results demonstrated that the co-polymer Pluronic F127 or PDDA coatings yielded longer stable dispersions (up to 20 h) than single polymer PEG [276]. Encapsulation is another effective route for the promotion of safety and stability of IONP/metal components and the formation of a new structure (such as the rattle structure) [277], and inorganic material SiO_2_ is a universal material. The Fe_3_O_4_–Au hybrid nanocrystal encapsulated in a silica nanosphere was synthesized via reducing AuCl_4_^−^ and the preferential nucleation of Au at the Fe_3_O_4_ surface. Then Fe_3_O_4_ was selectively dissolved through a reductive process. As a result, a nanorattle structure consisting of a hollow or porous silica nanoshell and Au nanocrystals can be generated [278].

#### 3.6.4. Metal oxides and sulfides

More and more metal oxides or sulfides have been used to protect or functionalize IONPs, mainly because of the fantastic magnetic properties of IONPs and other unique physical or chemical properties of metal oxides and sulfides.

**Figure 19.**
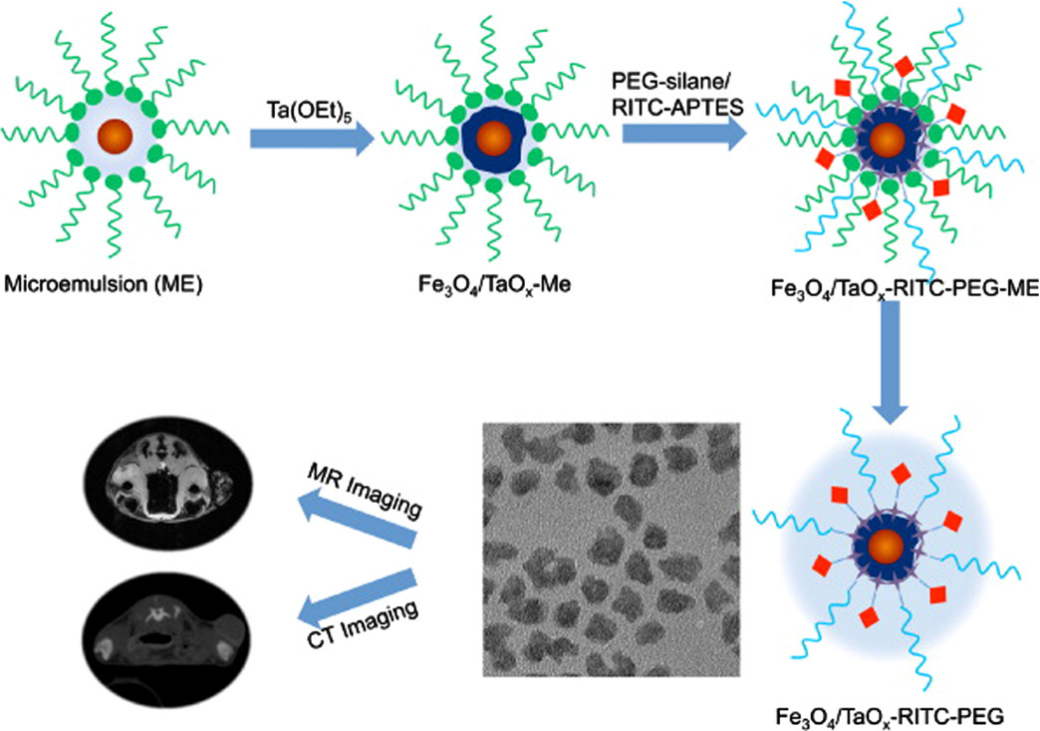
Schematic illustration of synthesis and modification of Fe_3_O_4_–TaO_*x*_ core–shell NPs, and application for simultaneous MRI and CT. (Reprinted with permission from N Lee *et al* 2012 *J. Am. Chem. Soc*. **134** 10309. Copyright 2012 American Chemical Society.)

Oxide and sulfide semiconductors are the most common compounds that are used to functionalize magnetic IONPs, such as TiO_2_ [279–281], ZnO [282], SnO_2_ [283, 284], WO_3_ [285], Cu_2_O [286], CdS [287–289], ZnS [290, 291], PbS [292], Bi_2_S_3_ [293], etc. For example, the spindle-like IONP@SnO_2_, IONP@TiO_2_ and IONP@ZnO composite NPs were synthesized successively by different wet-chemical routes; their composite NPs exhibited enhanced photocatalytic abilities for organic dyes, mainly owing to the synergistic effect between the narrow and wide bandgap semiconductors and effective electron-hole separation at the interfaces of iron oxides/semiconductors [281–284]. As shown in figure 19, Lee *et al* developed a sol–gel reaction of tantalum (V) ethoxide in a microemulsion containing Fe_3_O_4_ NPs that was used to synthesize multifunctional Fe_3_O_4_/TaO_*x*_ core–shell NPs recently, which were biocompatible and exhibited a prolonged circulation time. When the NPs were intravenously injected, the tumor-associated vessel was observed by using computed tomography (CT), and MRI revealed the high and low vascular regions of the tumor [294]. Wu *et al* have presented a very simple strategy for the synthesis of superparamagnetic and fluorescent Fe_3_O_4_–ZnS hollow nanospheres by a combining of corrosion and the Ostwald ripening process. These hollow nanospheres with diameters smaller than 100 nm are not only nontoxic with a highly porous shell but also exhibit very good magnetic resonance and fluorescence [295]. Indeed, semiconductors are common used as a functionalized layer to coat IONPs for obtaining bi-functional composite NPs, such as fluorescence and magnetic [212], photocatalyst and magnetic, etc.

**Figure 20.**
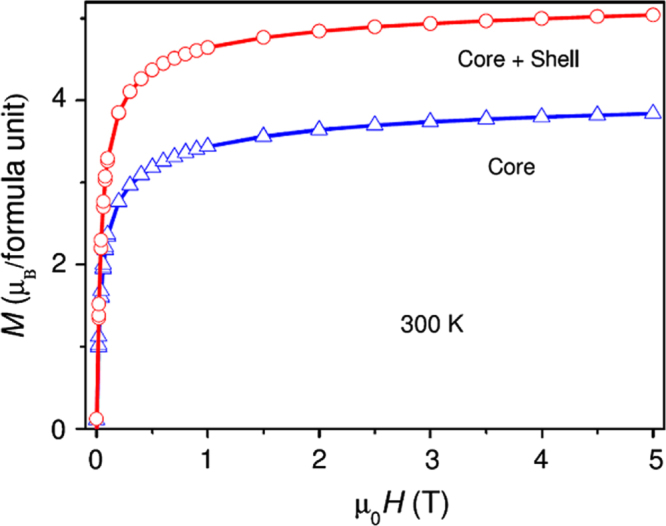
Magnetization versus field plots of bare core Fe_3_O_4_ and core–shell Fe_3_O_4_–*γ*-Mn_2_O_3_ NPs at 300 K. (Reprinted with permission from P Manna *et al* 2011 *J. Phys. Condens. Matter* **23** 506004. Copyright 2011 Institute of Physics.)

Magnetic materials coated on magnetic IONPs usually have a dramatic influence on the final magnetic properties, including the iron oxide itself [296], Co_3_O_4_ [297, 298], NiO [299], Mn_*x*_O_*y*_ [300, 301], CoFe_2_O_4_, MnFe_2_O_4_, etc. The combination of two different magnetic phases will generate new magnetic composites with many possible applications. For example, Manna *et al* have reported the magnetic proximity effect in a ferrimagnetic Fe_3_O_4_ core–ferrimagnetic *γ*-Mn_2_O_3_ shell nanoparticle system. As shown in figure 20, the magnetization of core–shell NPs is clearly greater than that of the bare core NPs [302]. Liu *et al* have developed the manufacture of a series of multifunctional magnetic core–shell hetero-nano-architectures (designated as Fe_3_O_4_@NiO and Fe_3_O_4_@Co_3_O_4_) by an *in situ* solvothermal-coating/decomposition approach. The resulting core–shell NPs presented a number of important characteristics, such as controllable shell thickness, excellent magnetism, stable recyclability as well as a large surface-exposure area. By taking advantage of the high affinity of the metal ion on the shell surface toward biomolecules and rapid response toward an assistant magnet, the Fe_3_O_4_@NiO can be applied to magnetically separate His-tagged proteins from a cell lysate and efficiently enrich peptides with different molecular weights from complex sample systems for mass spectrometry analysis [303].

## 4. Biomedical applications

The biocompatibility and toxicity of IONPs are important criteria to take into account for their biomedical applications. Parameters determining biocompatibility and toxicity are the nature of the magnetically responsive component, and the final size of the composite particles including their core and the coatings (shell). Ideally, composite IONPs must also have a high magnetization so that their movement in the blood can be controlled with an external magnetic field until it is immobilized close to the targeted pathologic tissue [153]. Magnetic IONPs with a long blood retention time, biodegradability and low toxicity have emerged as one of the primary nanomaterials for biomedical applications *in vitro* and *in vivo*. Some biomedical applications require surface functionalized, especially core–shell type, magnetic IONPs.

### 4.1. *In vivo* applications

IONPs have a large surface area and can be engineered to provide a large number of functional groups for cross-linking to tumor-targeting ligands such as monoclonal antibodies, peptides, or small molecules for diagnostic imaging or the delivery of therapeutic agents [304]. Especially, the magnetic properties of IONPs can be used in numerous *in vivo* applications, which can be divided into three main groups: (i) magnetic vectors that can be directed by means of a magnetic field gradient towards a certain location, such as in the case of targeted drug delivery; (ii) magnetic contrast agents in MRI; and (iii) hyperthermia or thermoablation agents, where the magnetic particles are heated selectively by application of a high-frequency magnetic field [305].

#### 4.1.1. Targeted drug delivery

**Figure 21.**
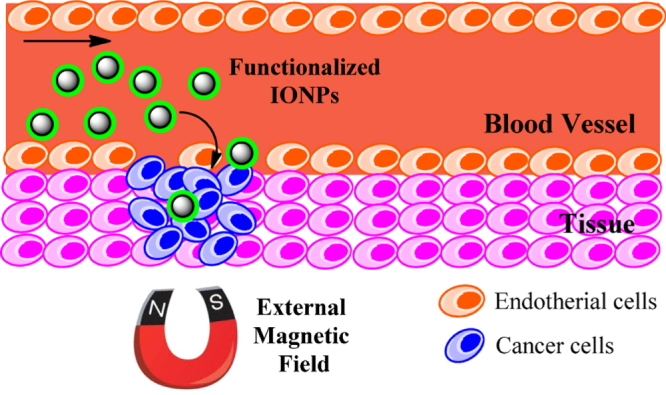
Schematic representation of magnetic nanoparticle-based drug delivery system: these magnetic carriers concentrate at the targeted site using an external high-gradient magnetic field. After accumulation of the magnetic carrier at the target tumor site *in vivo*, drugs are released from the magnetic carrier and effectively taken up by the tumor cells.

In traditional drug delivery systems, such as oral ingestion or intravascular injection, the medication is distributed throughout the body through systemic blood circulation. For most therapeutic agents, however, only a small portion of the medication reaches the affected organ and there is reduced drug diffusion through biological barriers causing a high incidence of adverse effects. Targeted drug delivery (TDD) seeks to concentrate the medication in the tissues of interest while reducing the relative concentration of the medication in the remaining tissues and crossing the biological barriers by active accumulation or an active targeting strategy [306]. Furthermore, magnetic IONP-based drug targeting (figure 21) is a promising cancer treatment method for avoiding the side effects of conventional chemotherapy by reducing the systemic distribution of drugs and lowering the doses of cytotoxic compounds [307]. Functionalized IONPs as a carrier can deliver a wide range of drugs to all areas in the body. Hence the efficient intracellular delivery of NPs is one of the main factors in enhancing the efficacy of the encapsulation therapeutic agent. Generally, magnetic IONPs are used as the core and biocompatible components act as a functionalized shell to form the core–shell structure for TDD carriers, and the drugs are bound or encapsulated into the polymer matrix. In a drug carrier system, the sizes, surface properties, and stability are the crucial features. Partially, the IONPs should be small enough to penetrate through the capillary bed. However, if the diameter of the IONPs is smaller than 10 nm, they will be rapidly removed through extravasations and renal clearance. Therefore, IONPs with a diameter ranging from 10 to 100 nm are optimal for intravenous injection and have the most prolonged blood circulation times [68]. Additionally, IONPs with a positive charge are better internalized by human breast cancer cells than those IONPs with negative charge. However, intake of these IONPs also depends upon cell type. IONPs with a hydrophobic surface are easily adsorbed at the protein surface and show a low circulation time [308]. Hence, many biocompatibility materials have been used to functionalize IONPs for TDD, such as biocompatible organic polymers (PEG, chitosan, dextran, etc), liposomes, silica, and bioceramics [182].

Nanostructure-mediated TDD, a key technology for the realization of nanocarriers, has the potential to enhance drug bioavailability, improve the timed release of drug molecules, easily functionalize with targeting ligands, and enable precision drug targeting (sensitivity to an external magnetic field). In particular, magnetic composite IONPs are currently recognized as one of the most promising modalities of such drug carriers [309–311]. Furthermore, nanostructure targeted IONPs carriers can not only serve as a vehicle for drug delivery, but also for gene delivery [312]. For instance, Qiu *et al* have developed an MRI visible gene delivery system Stearic-LWPEI-SPIO for the delivery of minicircle DNA (mcDNA). These Stearic-LWPEI-SPIOs possess a high mcDNA binding capability for the protection of mcDNA from enzymatic degradation and the controlled release of mcDNA in the presence of polyanionic heparin. Furthermore, Stearic-LWPEI-SPIO NPs loaded with mcDNA can enhance expression of luciferase in MCF-7 cells without evidently exhibiting cellular toxicity [313].

A high drug loading efficiency and drug-release rate are essential parameters for therapy, while mesoporous functional layers (such as SiO_2_ and C) and IONPs with hollow or mesoporous structure are enabling the promotion of drug loading [314, 315]. For example, Zhang *et al* reported the development of a magnetic drug carrier composed of doxorubicin-conjugated Fe_3_O_4_ nanoparticle cores and a PEG-functionalized porous silica shell (Fe_3_O_4_–DOX/pSiO_2_–PEG). The DOX loading capacity of the porous drug carrier system is 16.3 *μ*g mg^−1^. Fe_3_O_4_–DOX/pSiO_2_–PEG NPs can be internalized by cells through an endocytosis process, and can also be easily functionalized with a targeting ligand via a silicone coupling agent for increased and specific uptake of the drug carrier in tumor cells over-expressing the folate receptor, such as MCF-7 and HeLa cells [316]. Recently, Kayal and Ramanujan have reported the DOX loading and release profiles of PVA coated IONPs, which showed that they can release 45% of the adsorbed drug in 80 h; the results illustrated that composite NPs are promising magnetic drug carriers to be used in magnetically targeted drug delivery [317].

Moreover, carriers comprising coated magnetic IONPs loaded with an anti-cancer drug are injected into the patient’s body via the human circulatory system. An external magnetic field is used to localize the drug loaded carriers at the target site and the drug can then be released from the carriers either via enzymatic activity or changes in physiological conditions such as pH, osmolality, or temperature, and be taken up by target cells. Smart control release systems have been developed to meet the changed physiological conditions, enhance accumulation, and control drug release at the intended sites. The stimulus pH-/thermo-/photo-magnetic TDD systems are the common smart control release systems [318]. Generally, the pH of body tissue is maintained around 7.4 in a healthy human. However, there exist several mechanisms to modulate pH inside the body. The gastrointestinal tract changes the pH along the tube, which is ∼1–3 in the stomach and ∼7 in the intestine. Each mechanism has been used as a triggering signal for pH-responsive TDD. Basically, ionizable moieties, such as carboxylic acid, amine, azo, phenylboronic acid, imidazole, pyridine, sulfonamide, and thiol group functionalized IONPs can afford pH-sensitivity drug loaded carriers [319]. A thermosensitive polymer obtained by simple aliphatic modification of biocompatibility/biodegradable block copolymers exhibited a low critical solution temperature of ∼38 °C, thus the functionalized IONPs released the drugs in response to a variation in external temperature. For example, Zhang *et al* have successfully developed a novel magnetic drug-targeting carrier consisting of encapsulated magnetic IONPs with a smart thermosensitive polymer dextran-g-poly (N-isopropylacrylamide-co-N, N-dimethylacrylamide). The smart stimuli-responsive polymer enabled controllable drug release through small changes of temperature in the vicinity of a lower critical solution temperature (LCST) and pH. The system has a lower drug release at 20 °C (below the LCST), while at 40 °C (above the LCST) and at 37 °C (LCST) the drug release is high and rapid for the initial 5 h, followed by a sustained release at longer duration. The drug release is primarily influenced by a triggered drug release mechanism. The acidic medium favors drug release because of the acid-labile linker [320]. Additionally, targeted delivery of therapeutic agents to the brain has enormous potential for the treatment of several neurological disorders such as Alzheimer’s disease and brain tumors. However, the blood–brain barrier (BBB) significantly impedes the entry of drug molecules into the brain from the bloodstream. IONP-based targeted delivery represents a promising alternative strategy in overcoming the BBB [321].

Presently, the development of multifunctional magnetic IONPs which were coated or functionalized with polymers, lipidic, or inorganic shells further boost the potential use in medicine. These multifunctional IONPs not only serve as a vehicle for TDD but also have applications in MRI [322], targeted thermosensitive chemotherapy [323], and fluorescent/luminescence imaging [324]. For instance, Chourpa *et al* have developed novel biocompatible nanosystems, which are useful for cancer therapeutics and diagnostics (theranostics). These multifunctional NPs (SPION–DOX–PEG–FA) are not only for bimodal cancer cell imaging (by means of MRI and fluorescence) but also for bimodal cancer treatment (by targeted drug delivery and by the hyperthermia effect). Comparing the incubated cells with suspensions of SPION–DOX–PEG–FA and SPION–DOX–PEG shows they provide the same DOX fluorescence intensity. However, the uptake of the FA-functionalized NPs was twice that of SPION–DOX–PEG after 120 min of cell culture, while qualitatively the NP distribution remained similar and appeared as cytosolic staining, indicating that the quantity of FA grafted on the elaborated NPs appears sufficient to significantly increase their uptake by MCF-7 cancer cells [202]. Although considerable achievements have been reached in magnetic TDD systems, to date, actual clinical trials are still problematic. IONPs may be considered to be biocompatible, but the immune response during the residence time of the carrier–drug conjugate, the toxicity of carrier materials and their possible decomposition products still warrant further investigation.

#### 4.1.2. MRI

MRI is an imaging technique used primarily in medical settings to produce high quality images of the inside of the human body, which is based on the principles of nuclear magnetic resonance (NMR). Ultra small and related functionalized IONPs possess unique superparamagnetic properties, which generate significant susceptibility effects resulting in strong *T*_2_ (spin–spin relaxation process) and *T*_2_∗ contrast, as well as *T*_1_ effects (spin–lattice relaxation process) at very low concentrations for MRI, and are widely used for clinical oncology imaging as contrast agents [325–327]. A major barrier in the use of nanotechnology for practical applications is the difficulty in delivering NPs to intracranial positions. However, IONPs can deliver to the targeted site by external magnetic field. Additionally, a cellular magnetic-linked immunosorbent assay (C-MALISA) has been developed as an application of MRI for *in vitro* clinical diagnosis: first the different antibodies or fragments directed to several types of receptors can couple onto the surface of IONPs, then they can form a specific bind with the tumor for *in vivo* testing and magnetomotive optical molecular imaging [328]. Therefore, MRI is one of the most promising applications for magnetic IONPs [153].

Recently, Hadjipanayis *et al* reported IONPs (10 nm in core size) conjugated to a purified antibody that selectively binds to the epidermal growth factor receptor (EGFR) deletion mutant (EGFRvIII) present on human glioblastoma multiforme (GBM) cells and used for therapeutic targeting and MRI contrast enhancement of experimental glioblastoma, after convection-enhanced delivery. The MRI results revealed that a significant decrease in glioblastoma cell survival was observed after NP treatment and no toxicity was observed with treatment of human astrocytes (*P* < 0.001) [329]. Basly *et al* grafted small dendritic molecules through a phosphonate anchor by covalent attachment in order to stabilize iron oxide suspensions. The enhancement contrast ratio values are 15% to 75% higher than those obtained for Endorem^™^ (a commercial IONP contrast agent) on a MR *T*_2w_ image at 7 T. Such hybrid and biocompatible nanoscale objects may open a new route for the development of highly relaxing contrast agents displaying a quite satisfactory *R*_2_/*R*_1_ ratio even at high field [330].

**Figure 22.**
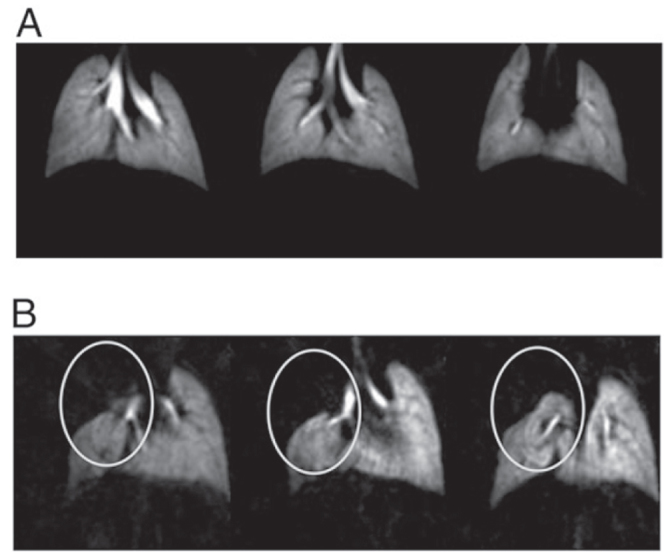
Detection of pulmonary metastases in a breast adenocarcinoma mouse model. (a) HP images (TE = 4 ms) from a control mouse, showing normal ventilation patterns. (b) Images from a human breast adenocarcinoma mouse model (TE = 4 ms) after injection of LHRH-SPIONs. A clear signal defect can be seen in the right lobe (yellow circles). All of the HP 3He lung MR images are formatted with 1 mm slice thickness. (Reprinted with permission from R T Branca *et al* 2010 *Proc. Natl Acad. Sci.* **107** 3693.)

*In vivo* cell tracking or labeling by MRI can provide the observation of biological processes and monitor cell therapy directly, which is another successful application of IONPs in MRI [331–333]. MRI allows for cell tracking with a resolution approaching the size of the cell when the cell loaded enough magnetic IONPs (for increasing the iron concentration). As shown in figure 22, Branca *et al* used cancer-binding ligand functionalized IONPs to target the cancer cells, then imaged by high-resolution hyperpolarized ^3^He MRI. *In vivo* detection of pulmonary micrometastates was demonstrated in mice injected with breast adenocarcinoma cells. This method not only holds promise for cancer imaging but more generally suggests a fundamentally unique approach to molecular imaging and cell tracking in the lungs [334]. Zhang *et al* investigated the feasibility of imaging green fluorescent protein (GFP)-expressing cells labeled with IONPs with the fast low-angle positive contrast steady-state free precession (FLAPS) method and to compare them with the traditional negative contrast technique. The GFP cell was incubated for 24 h using 20 *μ*g Fe mL^−1^ concentration of SPIO and USPIO NPs. The labeled cells were imaged using positive contrast with FLAPS imaging, and FLAPS images were compared with negative contrast *T*_2_∗-weighted images. The results demonstrated that SPIO and USPIO labeling of GFP cells had no effect on cell function or GFP expression. Labeled cells were successfully imaged with both positive and negative contrast MRI. The labeled cells were observed as a narrow band of signal enhancement surrounding signal voids in FLAPS images and were visible as signal voids in *T*_2_∗-weighted images. Positive contrast and negative contrast imaging were both valuable for visualizing labeled GFP cells [335].

The use of these IONP colloids as specific contrast agents for MRI is now a well-established area of pharmaceutical development. Feridex^®^, Endorem^™^, GastroMARK^®^, Lumirem^®^, Sinerem^®^, Resovist^®^ and more pending patents tell us that the last word in this area has not been said. There are three remarkable advantages of IONPs in MRI applications as follows: (1) a minimum delay of about 10 min between injection (or infusion) and MR imaging extends the examination time; (2) cross-section flow void in narrow blood vessels may impede the differentiation from small liver lesions; and (3) aortic pulsation artifacts become more pronounced [336].

It is noteworthy that more functionalized IONPs have been utilized in MRI [337–339]. For example, Smolensky *et al* have demonstrated that the incorporation of a thin organic coating containing efficient iron oxide and gold chelators significantly increased the saturation magnetization of core–shell iron oxide@gold NPs. The resulting high relaxivity of the nanocomposites, together with the small, compact structure of the assemblies and their characteristic plasmonic behavior, renders Fe_3_O_4_@organic@gold an attractive alternative to current magnetoplasmonic agents for multimodal cell imaging [340]. Recently, Zhou *et al* have prepared nearly monodispersed yolk-type Au@Fe_3_O_4_@C nanospheres with hollow cores of 50 nm in diameter through coating Au@SiO_2_ NPs with Fe_3_O_4_@C double layers, followed by dissolving SiO_2_. The coexistence of Fe_3_O_4_ and Au also makes the nanospheres dual probes for MRI with a specific relaxivity (*r*_2_∗) of 384.38 mM^−1^ s^−1^ and optical fluorescence imaging using a near-infrared excitation [341].

#### 4.1.3. Magnetic hyperthermia and thermoablation

**Figure 23.**
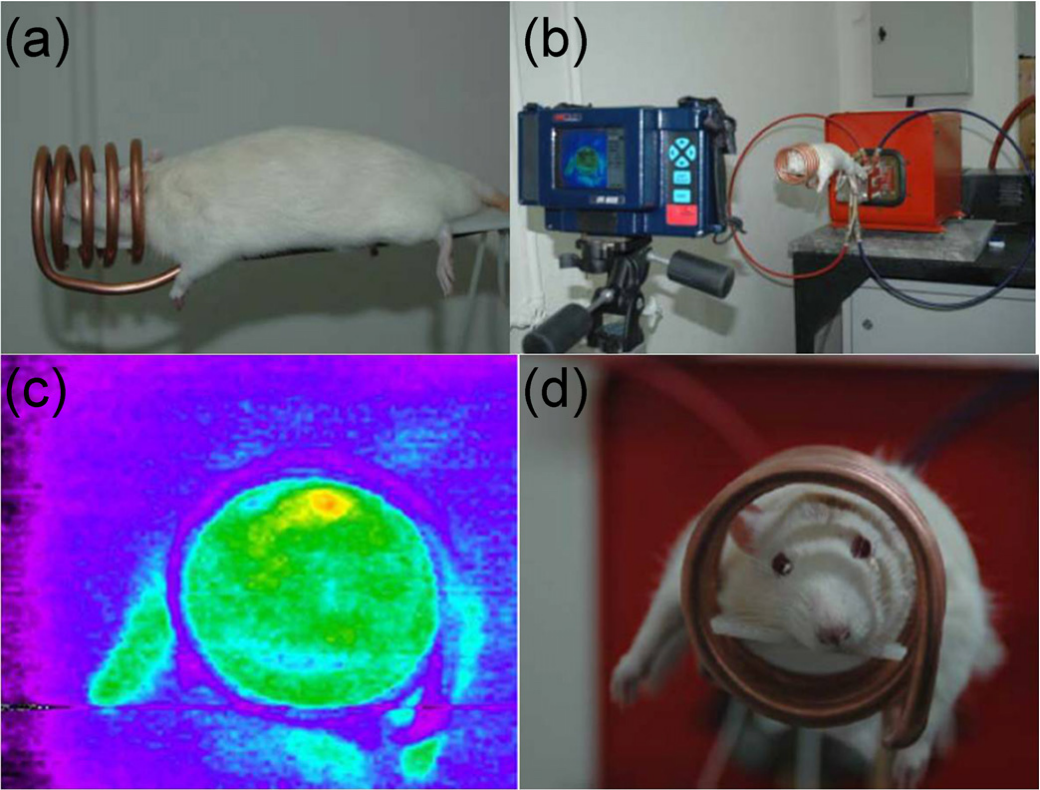
Ferrofluids containing IONPs are synthesized and characterized as possible agents for medical treatment and diagnosis. Specifically, novel iron-oxide-based NPs are investigated (i) as contrast agents for MRI, and (ii) for tumor treatment using the technique of magnetic hyperthermia where magnetic NPs are injected in the tumor and heated by applying a strong ac magnetic field. In the left picture the temperature increase of an extracranial tumor, after injecting a small quantity of ferrofluid and irradiating with low frequency radiofrequency waves (150 kHz).

The use of ferromagnetic NPs for magnetic hyperthermia and thermoablation therapies has attracted considerable attention as one of the promising treatments for cancer [342, 343]. Hyperthermia is the heating of cells in the range of 41–47 °C, which causes the preferential death of tumor cells [344]. When magnetic IONPs are subjected to an alternating magnetic field, heat generation is a result of a combination of internal Néel fluctuations of the particle magnetic moment, hysteresis, and the external Brownian fluctuations that all rely on the magnetic properties of IONPs. However, the temperature of the thermoablation method is often greater than 47 °C, which causes the rapid death of tumor cells due to the high temperature. Thus, some difficulties are faced when heating the tumor part to a sufficiently high temperature while simultaneously maintaining the normal tissues at a lower temperature. Modern techniques generally employ localized hyperthermia for cancer therapy [345]. Many techniques have been developed for the localized heating treatment of cancers, for example, radio-frequency waves (as shown in figure 23 [346]), microwaves and ultrasounds [347–349].

In general, the specific adsorption rate (SAR) is the main parameter determining how effectively NPs generate heat to the tissue during magnetic hyperthermia treatment. SAR is the rate at which electromagnetic energy (*E*_em_) is absorbed by a unit mass of a biological material (*m*) when exposed to a radio frequency (*R*_F_) electromagnetic field.

It can be expressed as follows:
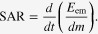



For a ferrofluid sample, the SAR is usually averaged either over the whole body, or over a small sample volume (typically 1 g or 10 g of tissue). It is also related to the electric field or the temperature rise at a given point [350], and hence it can be calculated from the electric field within the tissue as follows,
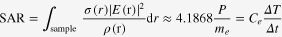
where *σ* is the sample’s electrical conductivity, *E* is the RMS electric field, *ρ* is the sample density, *P* is the electromagnetic wave power absorbed by the sample, *m*_*e*_ is the mass of the sample, and *C*_*e*_ is the specific heat capacity of the sample. Therefore, SAR can expressed as being proportional to the rate of the temperature increase (*ΔT*/*Δt*). Furthermore, according to the equation, for both the ferrofluid sample and the human body, the conductivity can be zero. Generally, SAR is commonly used to measure power absorbed from mobile phones and during MRI scans. The value will depend heavily on the geometry of the part of the body that is exposed to the RF energy (low-frequency magnetic wave of 100–400 KHz), and on the exact location and geometry of the RF source.

Hence, increased heating rates of magnetic IONPs are an important challenge in order to minimize dosages of magnetic IONPs needed to reach therapeutic temperatures in magnetic hyperthermia or thermoablation. Possible approaches to increase heating rates are increasing the anisotropy of the NPs (shape or magnetocrystalline) or increasing the field strength. An alternative approach to increase the heating rates would be to increase the monodispersity of a sample of magnetite NPs. Gonzales-Weimuller *et al* reported the size-dependant heating rates of IONPs for magnetic fluid hyperthermia. The results demonstrated that the SAR does indeed vary with particle size. The highest SAR measured was 447 W g^−1^ at 24.5 kA m^−1^ for 11.2 nm particles and models, indicating that higher heating rates were possible by increasing the size of the particles to 12.5 nm [351].

**Figure 24.**
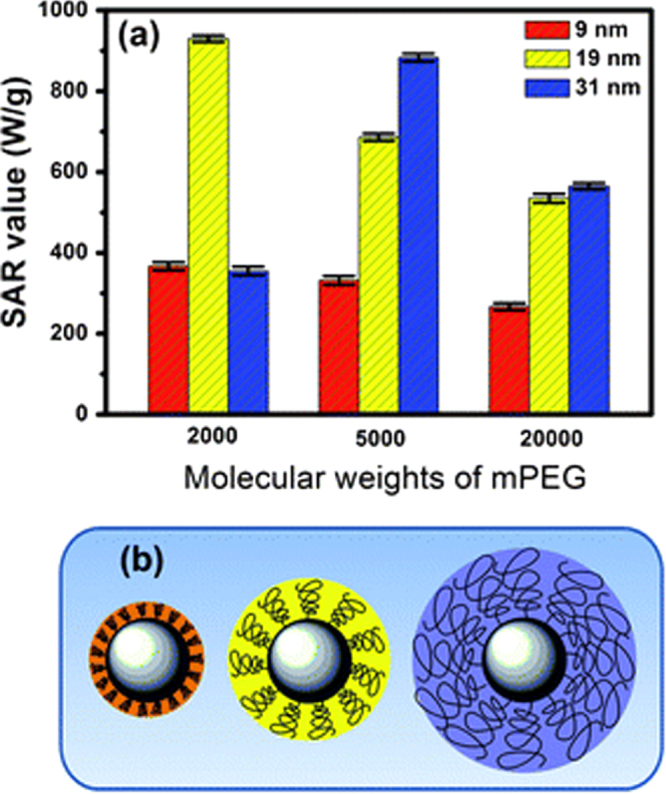
(a) The SAR values of the different sized Fe_3_O_4_ NPs for different mPEG: 9 nm (orange), 19 nm (yellow), 31 nm (blue). (b) A schematic diagram of nanoparticle based hyperthermia agents with iron oxide core and varied mPEG coating. (Reprinted with permission from X L Liu *et al* 2012 *J. Mater. Chem.* **22** 8235. Copyright 2012 Royal Society of Chemistry.)

Some reports reveal that the surface functionalization improved the hyperthermic effect. As shown in figure 24, Liu *et al* reported that enhanced SAR with decreased surface coating thickness was observed and ascribed to the increased Brownian loss, improved thermal conductivity as well as improved dispersibility [352]. Moreover, the inorganic coating can also improve the SAR value. For example, Mohammad *et al* found that the hyperthermic effect of SPIONs is enhanced dramatically on coating with Au. The results indicated possibilities for utilization of very low frequency oscillating magnetic fields in hyperthermia treatment. The gold coating should retain the superparamagnetic fraction of the SPIONs much better than when compared to uncoated SPIONs alone; this leads to a higher energy of magnetic anisotropy of superparamagnetic NPs within the gold shell as compared to uncoated SPIONs. Excellent hyperthermia exhibited by SPION@Au NPs coupled with their lack of cytotoxicity was anticipated to make them into suitable candidates for thermolysis of cancer cells [353]. Recently, Balvata *et al* reported their magnetic hyperthermia results obtained after intratumoral injection, demonstrating that micromolar concentrations of iron given within the modified core–shell Fe/Fe_3_O_4_ NPs caused a significant anti-tumor effect on murine subcutaneous mouse melanoma with three short 10 min alternating magnetic field (AFM) exposures. These results indicated that intratumoral administration of surface modified MNPs attenuated mouse melanoma after AMF exposure, and these MNPs were capable of causing an anti-tumor effect in a mouse melanoma model after only a short AMF exposure time [354].

SPIONs can be considered as a very promising agent for hyperthermia therapy, but this new field of application requires an improvement of the reproducibility, size and shape controlling in the preparing process and its biocompatibility. Moreover, how to apply the cancer treatment of fine tissues (such as brain and kidney) is also an ongoing challenge.

### 4.2. *In vitro* applications

Another important kind of application of functionalized IONPs is *in vitro* application (such as biosensor, cell bioseparation), which promises increased sensitivity, speed, and cost-effectiveness. There are several promising nanocomposites for *in vitro* application, such as Au NPs, and quantum dots (QDs). There are already *in vitro* diagnostic products on the market, based on magnetic IONPs.

#### 4.2.1. Bioseparation

**Figure 25.**
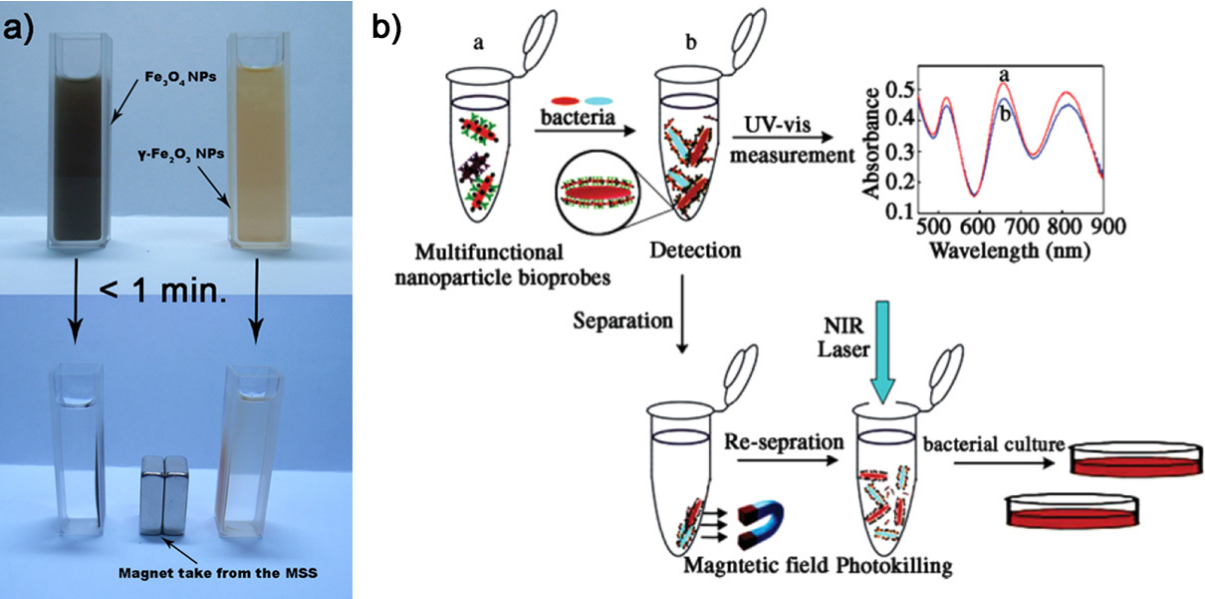
(a) The Fe_3_O_4_ and *γ*-Fe_2_O_3_ hollow NPs (dispersed in ethanol solution) before and after magnetic separation by an external magnet. (b) The detection, separation, and thermal ablation of multiple bacterial targets. (Reprinted with permission from C G Wang and J Irudayar 2010 *Small* **6** 283. Copyright 2010 John Wiley and Sons.)

As a successful application of magnetic IONPs, bioseparation is also an important kind of application, especially for *in vitro* DNA, antibody, protein, gene, enzyme, cell, virus and bacteria separation [355–358]. Compared with the traditional separation procedures, magnetic separation has many advantages of being able to be quickly localized or retrieved with a common magnet (as shown in figure 25(a)) [18], which is faster and more cost-effective than traditional column affinity chromatography. Generally, surface functionalized magnetic IONPs with suitable intermediates are commonly used to enhance the separation efficiency with such modification of surfactants, polymers, and ligands for introducing functional end groups (such as –OH, –NH_2_, –SH, –COOH, etc) through the selective adsorption to the target biomolecules [359].

Recently, Chang *et al* reported a novel approach to efficiently separate proteins such as bovine serum albumin (BSA) by the modification of hydrophobic pockets on the surface of Fe_3_O_4_@SiO_2_ NPs with various alkyl groups at various pH levels. The magnetic separation efficiency was strongly reflected and could be attained by controlling the size of the hydrophobic pocket and other factors such as the alkyl chain length, salt concentration, and pH levels [360]. Wang and Irudayaraj described the development of a facile route to the site-selective assembly of Fe_3_O_4_ NPs onto the ends, or ends and sides, of gold nanorods with different aspect ratios to create multifunctional nanorods incorporating optical and magnetic materials that provided tunable plasmonic and magnetic properties. As shown in figure 25(b), the Fe_3_O_4_–Au necklace-like hybrid NPs were functionalized with relevant antibodies to construct efficient platforms for simultaneous optical detection, magnetic separation, and thermal ablation of multiple pathogens from a single sample [361].

Additionally, IONP-based magnetic separation applications involve strict requirements such as chemical composition, particle size and size distribution, stability of magnetic properties, morphology, adsorption properties and low toxicity, and so on. For instance, Reza *et al* found that the BSA protein adhesions for magnetite–silica, magnetite–aminosilane and magnetite–silica–aminosilane arrays were 12.5%, 79.5% and 145.75% higher than for pure magnetite, respectively [362]. Arsianti *et al* systematically investigated the effect of IONP–PEI–DNA arrangement on transfection efficiency by varying the vector component mixing order. The aim was to elucidate the role of IONPs and PEI in gene delivery and to gain a fundamental understanding of the design of a suitable IONP-based vector for optimal plasmid DNA transfection. The highest magnetic vector cellular uptake was observed for the largest IONP vector (IONPs + PEI/DNA) due to enhanced gravitational and magnetic aided sedimentation onto the adherent cells. The highest gene expression was also observed for this MNP vector configuration [363].

Magnetic IONPs with a core–shell structure may enable this development of bioseparation fields, especially silica coated IONPs [364–366]. For example, Shao *et al* have reported three-component microspheres containing a SiO_2_-coated Fe_3_O_4_ magnetite core and a layered double hydroxide (LDH) nanoplatelet shell via an *in situ* growth method. The microspheres possess superparamagnetism and high saturation magnetization (36.8 emu g^−1^), which allows their easy separation from the solution, by means of an external magnetic field, and subsequent reuse. The microspheres show highly selective adsorption of the His-tagged protein from *Escherichia coli* lysate, demonstrating their utility [367]. The collection and separation rates for targets in a complex environment are crucial in bioscience.

#### 4.2.2. Biosensor

Biosensing as an effective diagnostic platform has been developed to detect biomolecules and cells with high sensitivity that could enable early disease diagnosis [368]. Magnetic nanosensors exhibiting high specificity and biocompatibility have been synthesized for the *in vitro* and *in vivo* detection of molecular interactions. Upon target-induced nano-assembly formation, a sensitive and dose-dependent decrease in the spin–spin relaxation time (*T*_2_, can be detected by magnetic resonance (NMR/MRI) techniques) of adjacent water [369]. Furthermore, the superparamagnetic IONP core of an individual nanoparticle becomes more efficient at dephasing the spins of surrounding water protons, enhancing *T*_2_ relaxation times so that the NPs act as magnetic relaxation switches (MRS) in the cooperative assembly process. For example, Perez *et al* found that the MRS nanosensor can detect specific mRNA, proteins, enzymatic activity, and pathogens (e.g., a virus) with sensitivity in the low femtomole range (0.5–30 fmol) [370, 371]. In addition, the anisotropy of the magnetic shape was used to control the critical applied magnetic field required to switch the magnetization of the element between its two stable states, thus creating a binary barcode.

**Figure 26.**
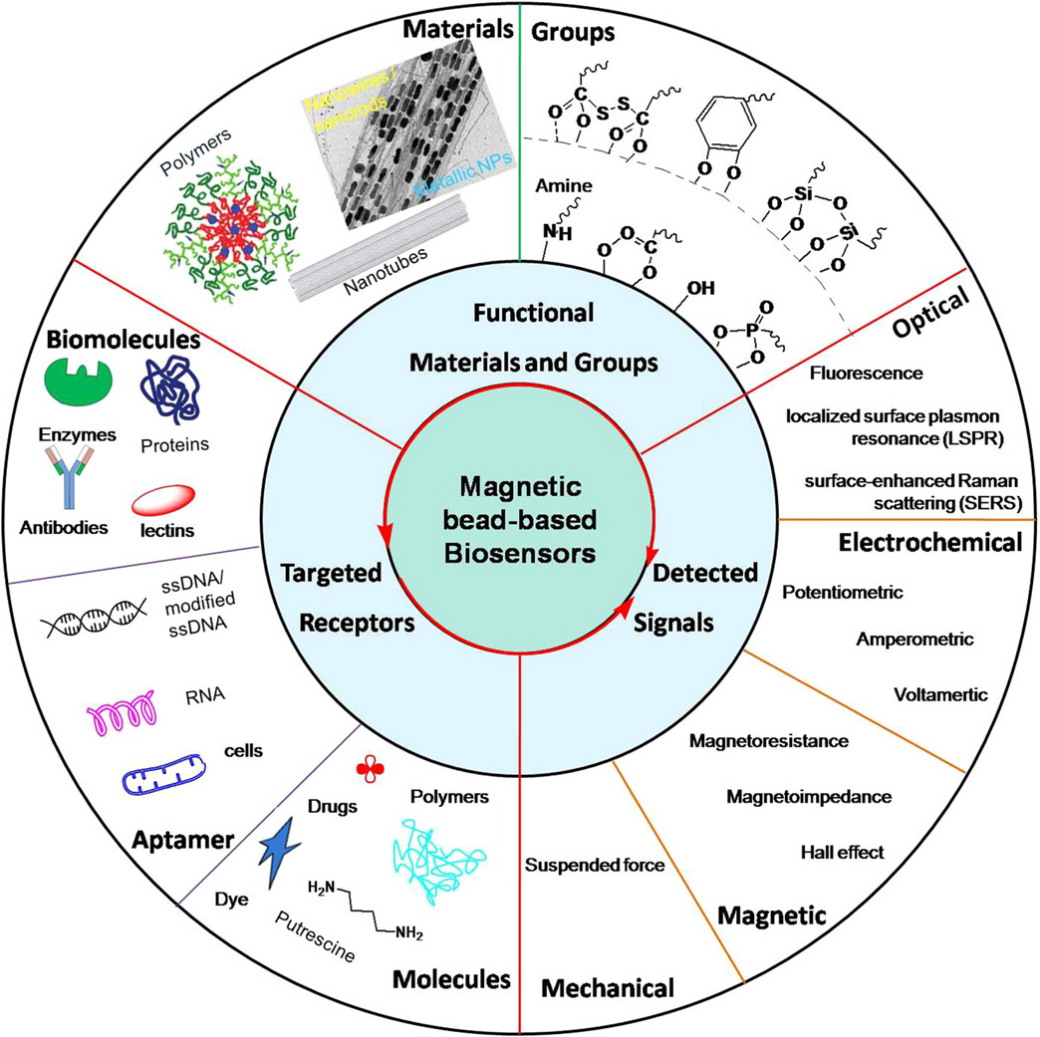
The components for magnetic biosensor and detail information.

However, to date, magnetic biosensors for diagnosis have not only been based on the properties of IONPs, but also on functionalized coated materials. The magnetic bead-based biosensors are functionalized IONPs by conjugating targeting ligands, which endow new specificity to the magnetic bead–based biosensors. Simultaneously, the IONPs, together with targeted receptors and functionalized layers/materials, act as the generator or detector of a signal, assigning the sensitivity of magnetic bead-based biosensors (figure 26). Generally, optical-magnetic bead-based biosensors exhibit excellent optical performance because of the unique interactions between light waves and the surface coating materials (such as Au, Ag, QDs and fluorescent molecules), which displayed excellent localized surface plasmon resonance (LSPR), surface-enhanced Raman scattering (SERS), and fluorescence [372, 373]. One-dimensional nanostructures (such as carbon nanotube, nanowires, and grapheme) have been applied as functional components for electrochemical magnetic bead-based biosensors [374, 375]. Those are usually based on a field-effect transistor (FET) where analyte molecules act as a gate, which controls the electrical resistance by causing depletion or accumulation of charge carriers. And the electrochemical–magnetic bead-based biosensors in clinical diagnosis are based on glucose, lactate, cholesterol, urea, creatine, and creatinine biosensors [376–378].

Many studies have been conducted on developing all kinds of magnetic bead-based biosensors with high-sensitivity and high-specificity, which have opened up an era of early disease diagnosis and better treatment [379, 380]. A composite electrode of glucose oxidase (GOD)–Fe_3_O_4_–Cs–Nafion was developed for glucose by combining the intrinsic peroxidase-like activity of Fe_3_O_4_ NPs and the anti-interference ability of the Nafion film. The novel glucose biosensor showed a relatively rapid response, high sensitivity (11.54 *μ*Acm^−2^ mM^−1^), low detection limit (6 × 10^−6^ M) and broad linear range (from 6 × 10^−6^ to 2.2 × 10^−3^ M). Furthermore, the wide detection range and high sensitivity may be assigned to the amplification of the magnitude of the current response for catalysis of H_2_O_2_ by Fe_3_O_4_ NPs [381]. Composite immunosensors based on gold NPs (GNPs), Fe_3_O_4_ NP–functionalized multiwalled carbon nanotube–chitosan (Fe_3_O_4_–FCNT–CS) and BSA composite film, were developed for the determination of carbofuran. The immunosensors exhibited good accuracy, high sensitivity, and stability for the detection of carbofuran. Under optimal conditions, the current detection limit was proportional to the concentration of carbofuran ranging from 1.0 ng mL^−1^ to 100.0 ng mL^−1^ and from 100.0 ng mL^−1^ to 200 *l*g mL^−1^ with the detection limit 0.032 ng mL^−1^ [382].

Importantly, the sensitivity and specificity of magnetic biosensors are the crucial indicators to evaluate the newly developed magnetic biosensors for diagnosis. Furthermore, continuously improving the sensitivity and specificity of magnetic biosensors is a requirement of analysis testing [383, 384]. The routes for improvement of the sensitivity and specificity of magnetic biosensors were adopted by the measurement signal amplification via enzymatic amplification [385], signal amplification by applying NPs [386] polymers combination with functional groups [387]. For instance, IONPs and a grating-coupled surface plasmon resonance (GC-SPR) sensor surface with metallic diffraction grating were modified with antibodies that specifically recognize different epitopes of the analyte of interest. Furthermore, the detection of *β* human chorionic gonadotropin (*β*hCG) was implemented to evaluate the sensitivity of the IONP-enhanced GC-SPR biosensor. The results revealed that the sensitivity of *β*hCG detection was improved by four orders of magnitude compared with the regular SPR sensor with direct detection format, and a limit of detection below pM was achieved [372].

Due to the complex components, and sensitivity and specificity requirements, the conventional routes for magnetic bead-based biosensors are a stepwise assembly process [388, 389], layer-by-layer assembly [390], and primary products that are solid-state platforms. Recently, however, research has demonstrated that solution-based platforms are beneficial over solid-state platforms because of the increasing probability of interaction between magnetic bead-based biosensors and analytes and allowing for biomolecule-based analytes to preserve their native form and function when in solution. For example, the lysine-modified diacetylene monomer with 10,12-pentacosadiynoic acid (Lys-PCDA)–SPION particles was prepared by the self-assembly of Lys-PCDA onto OA-SPIONs in the solution phase, which acted as a platform for the capture and detection of serum proteins. This magnetic biosensor showed a colorimetric response that corresponded to an increasing concentration of anti-BSA antibodies, with a lower detection limit of 0.5 mg mL^−1^ [391].

The present trend for magnetic bead-based biosensors is not only to connect the sensitivity and specificity but also to develop multiplexed magnetic bead-based biosensors for multi-detection [392]. There has been huge progress in the development of magnetic bead-based biosensors in recent years, but their application in clinical diagnosis is not common, except for glucose magnetic bead-based biosensors, which represents the large global market. There are still challenges for magnetic bead-based biosensors in clinical diagnosis [393].

## 5. Summary and perspectives

The size, size distribution, and shape of IONPs are the important parameters influencing the pharmacokinetic and bio-distribution *in vivo* applications [394]. However, absolute control over the shape and size distribution of magnetic IONPs remains a challenge, and the different formation mechanisms of iron oxides under different conditions still need to be investigated. Furthermore, the ligands and functional layers are often comparable in size to the IONPs, and their coupling effect can significantly increase the hydrodynamic size. Concequently, the increasing hydrodynamic diameter contributes to the macrophage and systemic clearance of functionalized IONPs. Therefore, it is necessary to measure the hydrodynamic diameter and zeta potential of IONPs before and after functionalization. Moreover, bio-distribution is also related to the IONP size and final colloid stability, which determines the fate of IONPs in *in vivo* and *in vitro* applications. Mitragotri and Lahann already concluded the size-dependent processes of NP transport in the human body [395], and the optimal hydrodynamic diameter range for *in vivo* application of intravenously injected NPs is 10–100 nm [396].

In addition, the surface properties IONPs such as surface charge play an important role in the physical stability and influence the interaction of IONPs with the biological system and their safety. In general, positively charged IONPs interacting strongly with blood components undergo relatively rapid clearance from systemic circulation, leading to nonspecific tissue uptake. In contrast, negatively and neutrally charged IONPs show lower interaction with plasma proteins than positively charged IONPs, and tend to nonspecifically stick to the cells [397]. Furthermore, studies on the influence of the thickness of the functional layer, dispersant packing density, and dispersant conformation on the protein resistance of sterically stabilized IONPs have to be designed carefully to make sure that the right outcomes are arrived at.

The saturation magnetization (*M*_s_) of uncoated and functionalized IONPs is one essential parameter that describes the magnetic response of IONPs. The *M*_s_ has been shown to decrease if the IONPs are satirically stabilized. However, *M*_s_ is increased by agglomeration of IONPs. Furthermore, the anchor chemistry can also affect *M*_s_ via strong interactions with the ions in the surface layer of the magnetic core [397, 398].

Though magnetic IONPs exhibit many unique properties which endow various advantages and opportunities in biomedical applications, more toxicological research is needed on the as-synthesized IONPs, and the criteria to evaluate toxicity need to be clearly defined [399, 400]. For future studies, the use of better and faster methods to improve our understanding of nanoparticle toxicity mechanisms should greatly advance the field [401]. Additionally, the biocompatibility of IONPs is linked to both the intrinsic toxicity of functional layers and its biodegradation metabolites, and to the immune system response following its administration. Importantly, when associated with functional layers, the toxicity profile of the IONPs may be increased or decreased as a consequence of the modification of their cell/tissue biodistribution and clearance/metabolization. Accumulation may, indeed, occur in biological sanctuaries where IONPs cannot diffuse when administered alone.

It is desirable, especially for research and application purposes, to add multifunctional labels or (imaging modalities), such as fluorophores or radiotracers to IONP surfaces. The successful engineering of multifunctional NPs would be of particular interest for the development of theranostic nanomedicine. However, the challenge remains in the clinical translation of nanoparticle probes, and issues such as biocompatibility, toxicity, *in vivo* and *in vitro* targeting efficiency, and long-term stability of the functionalized IONPs need to be addressed [402].
